# Sex-specific insights in atherosclerosis and pulmonary arterial hypertension: an overlooked comorbidity

**DOI:** 10.3389/fcvm.2026.1733624

**Published:** 2026-03-03

**Authors:** Jill Rose, Tiffany Chang, Thao Nghiem, Aashni Shah, Rushna Shaikh, Morgan Gardner, Kamilah Ali, Suellen D. Oliveira, Mabruka Alfaidi

**Affiliations:** 1 Department of Cellular & Integrative Physiology, University of Nebraska Medical Center, Omaha, NE, United States; 2 Vascular Immunobiology Lab, Department of Anesthesiology, College of Medicine, University of Illinois, Chicago, IL, United States; 3 Department of Physiology and Biophysics, College of Medicine, University of Illinois, Chicago, IL, United States; 4 Trinity College of Arts & Sciences, Duke University, Durham, NC, United States; 5 Department of Basic Sciences, Touro College of Osteopathic Medicine, Touro University, New York City, NY, United States; 6 Center for Heart and Vascular Research, University of Nebraska Medical Center, Omaha, NE, United States

**Keywords:** atherosclerosis, pulmonary arterial hypertension, sex chromosome, sex differences, sex Hormones

## Abstract

The mortality rates attributed to cardiovascular diseases (CVD) are increasing within the United States. Atherosclerotic cardiovascular disease (ASCVD) and pulmonary arterial hypertension (PAH) are two severe, life-threatening subtypes of CVD. Although ASCVD and PAH are distinct vascular disorders, they share common mechanisms, including endothelial dysfunction, inflammation, smooth muscle proliferation, fibrosis, and vascular remodeling. It is noteworthy that patients diagnosed with PAH may have underlying atherosclerotic coronary artery disease at a rate of ∼28%, and conversely, patients with ASCVD may present with pulmonary symptoms. PAH is more prevalent among females; however, once the disease is established, males exhibit disproportionately worse right ventricular (RV) adaptation and higher rates of RV failure. Conversely, atherosclerosis is more common in males and less prevalent in females before menopause. Despite advances in understanding the unique pathophysiology of each disease, the relationship between ASCVD and PAH remains poorly elucidated, and current animal models often fail to accurately replicate the complexities of both conditions. This review underscores the key similarities and differences between ASCVD and PAH, with particular emphasis on sex as a significant biological factor in these diseases. Recognition of these sex-specific vascular and cardiac mechanisms has important therapeutic implications, supporting sex-informed risk stratification and the development of targeted interventions for both pulmonary and systemic vascular diseases.

## 1 Introduction

**Figure 1:**
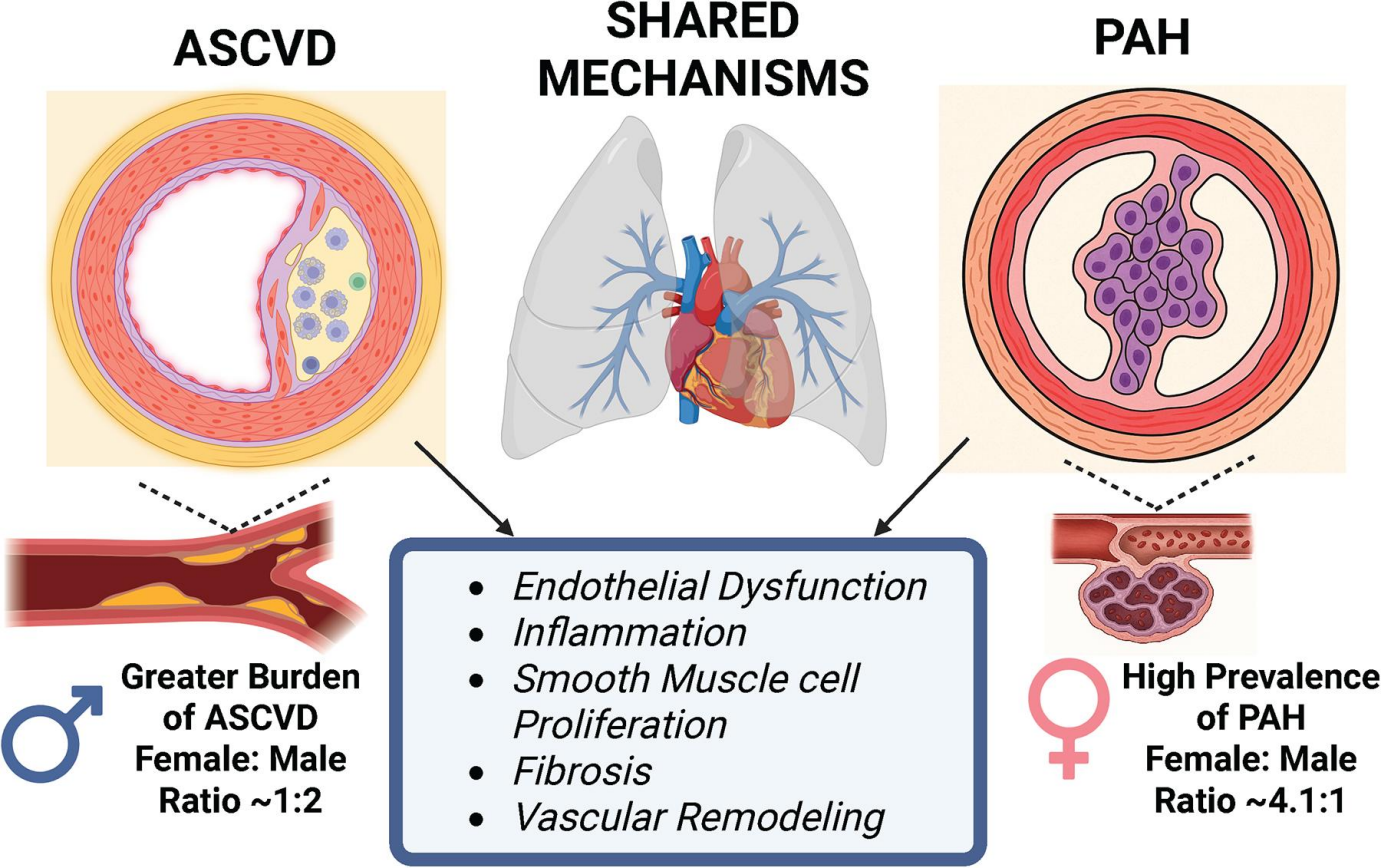
Schematic illustration of shared mechanisms between ASCVD and PAH, including endothelial dysfunction, inflammation, smooth muscle cell proliferation, fibrosis, and vascular remodeling. Arrows indicate shared disease mechanisms. PAH is more common in females than males (4.1:1 ratio) (10), while ASCVD is more prevalent in males than females (2:1 ratio) (11).*Generated in bioRender.

Atherosclerotic Cardiovascular Disease (ASCVD) and Pulmonary Arterial Hypertension (PAH) contribute to numerous forms of cardiovascular disease, one of the leading causes of death globally (1). ASCVD is characterized by the sudden occlusion of the main coronary arteries, leading to myocardial ischemia or infarction, resulting in right or left ventricular dysfunction and heart failure. PAH is characterized by elevated mean pulmonary arterial pressure, right ventricular hypertrophy (RVH), and right ventricular (RV) dysfunction, often culminating in RV failure. Both conditions are chronic and progressive, developing gradually throughout an individual's lifetime, and typically becoming evident only in advanced stages. Currently, both are incurable and associated with adverse health outcomes (2, 3). Atherosclerosis, the underlying disease process in ASCVD, is a chronic process characterized by endothelial dysfunction and ongoing inflammation, with infiltration of immune cells, primarily macrophages, into large and medium-sized arteries (4–6). PAH involves endothelial dysfunction, inflammation, vasoconstriction, and arteriolar remodeling (7, 8). Although the underlying pathology of PAH appears to be multifactorial, it involves cellular and molecular mechanisms also observed in atherosclerosis. In both ASCVD and PAH, excessive vascular inflammation and remodeling of the arterial wall ultimately led to sudden occlusion and heart failure (9). Figure 1 shows some shared vascular mechanisms between ASCVD and PAH.

### 1.1 Sex differences in prevalence, incidence, and mortality of ASCVD and PAH

Multiple extensive population-based studies consistently demonstrate that ASCVD is more prevalent in men until midlife, after which women's incidence sharply increases post-menopause. In the United States, men aged 40–59 years exhibit approximately 2.6-fold higher prevalence of ASCVD than women (95% CI 2.4–2.8) (1, 12). After age 60, this disparity significantly diminishes, and by age 75 and older, the prevalence in women slightly surpasses that in men (27.1% vs. 26.0%, RR 1.04, 95% CI 1.01–1.07) (13). Mortality rates are comparatively lower in premenopausal women but increase post-menopause, with a 52% rise in ASCVD-related mortality among women aged 75 years or older relative to men (HR 1.52, 95% CI 1.44- 1.4401.60) (14). Conversely, PAH exhibits an opposite sex distribution, with women accounting for approximately 65%–80% of idiopathic PAH cases across major registries such as COMPERA, REVEAL, and ASPIRE (15–17) (Table 1). Despite the higher incidence in women, they consistently demonstrate superior adjusted survival rates (see Table 1). These quantitative registry findings substantiate the sex paradox, wherein females demonstrate greater susceptibility to PAH yet exhibit better survival outcomes, thereby supporting the influential role of hormonal and molecular sex modifiers in the disease process.

**Table 1:** COMPREA, REVEAL, and ASPIRE Registry Information.

Registry	% Female	Female: Male Ratio	1-Year Mortality (Women vs. Men)	Adjusted Hazard Ratio for Death	Notes
COMPERA	65%	1:8:1	11% vs. 15%	0.78 (95% CI 0.65–0.93)	Better functional class at baseline in women (15).
REVEAL	79%	3:8:1	10% vs. 17%	0.75 (95% CI 0.63–0.88)	Women have lower BNP and higher DLCO (16).
ASPIRE	70%	2:3:1	15% vs. 27%	0.56 (95% CI 0.44–0.71)	Improvement persists after adjustment for age and etiology (17).

### 1.2 ASCVD and PAH may coexist in cardiovascular disease (CVD) patients

Pulmonary arterial medial hypertrophy and structural changes in distal arterioles have been observed in patients with advanced ASCVD, indicating that systemic vascular pathology extends beyond the coronary and systemic arterial beds (18). Histopathological and imaging studies suggest that atherosclerosis-related endothelial dysfunction and inflammation are not confined to the coronary or systemic arterial beds but can extend to the pulmonary circulation. Patients with advanced ASCVD exhibit pulmonary arterial medial hypertrophy, endothelial dysfunction, and distal arteriolar remodeling, even in the absence of overt left heart failure (19, 20). Systemic inflammation, oxidative stress, and circulating pro-atherogenic mediators can impair pulmonary endothelial nitric oxide (NO) signaling and promote smooth muscle cell proliferation in ASCVD. These pulmonary vascular changes often mirror those seen in PAH (5, 19–21), suggesting a common pathophysiological mechanism for ASCVD and PAH (Figure 1). Clinical studies further associate ASCVD with reduced pulmonary vascular compliance and early exertional dyspnea, suggesting subclinical pulmonary vascular involvement that precedes clinically apparent pulmonary hypertension (20, 21). This emerging link underscores the need of reevaluate how systemic atherosclerosis may predispose individuals to pulmonary vascular dysfunction, thereby blurring the traditional distinctions between right- and left–heart–related vascular diseases.

### 1.3 ASCVD and PAH common risk factors and underlying sex differences

Both ASCVD and PAH, although affecting different vascular areas, share several common risk factors such as hypertension, diabetes mellitus, smoking, obesity, obstructive sleep apnea (OSA), and chronic pulmonary or cardiac conditions (22–24). Elevated systemic blood pressure, abnormal metabolic profiles, and tobacco use promote vascular inflammation and dysfunction, leading to the formation of atherosclerotic plaques (25, 26). Similarly, factors like obesity, OSA, chronic pulmonary or cardiac diseases, and genetic predispositions also raise the risk of PAH (27, 28). Importantly, conditions like OSA serve as a connecting link, worsening systemic hypertension and cardiovascular stress, while also increasing pulmonary artery pressure through intermittent hypoxia and endothelial damage (29). This overlap highlights the potential interconnection of the mechanistic functions of the two diseases at the systemic, cellular, and molecular levels. Table 2 outlines the risk factors and sex differences linked to ASCVD and PAH.

**Table 2:** Risk Factors and sex differences between ASCVD and PAH.

Risk Factor/Aspect	Atherosclerosis (ASCVD)	Pulmonary Hypertension (PH/PAH)	Mechanisms Underlying Sex Differences
Age and disease onset	Post-menopause, women experience more rapid plaque instability due to histological changes, loss of smooth muscle cells, and altered calcification, resulting in a plaque burden similar to that of men in older age (30, 31).	Onset is generally between ages 30–60; females are affected more often (approximately 3–5 times more in females than males) (10).	Estrogen loss promotes endothelial dysfunction and adverse lipid/inflammatory shifts; in PAH, female predominance with better survival vs. men reflects sex differences in RV adaptation and hormone signaling (32, 33).
Traditional risk factors	Smoking increases ASCVD risk more sharply in women, up to a 25% higher risk of CAD relative to men. Diabetes and obesity also confer an especially elevated risk in women (34, 35).	BMI and airflow abnormalities (obstructive/restrictive patterns) are strongly associated with elevated pulmonary pressure in women; chronic lung disease relates more to elevated PASP in men (36).	Sex differences reflect divergent metabolic responses, adipose inflammation, and endothelial sensitivity to hyperglycemia and tobacco toxins. Women demonstrate greater microvascular dysfunction for equivalent risk exposure (37–39).
Unique female-disease-specific factors	Women face additional CVD risks from adverse pregnancy outcomes, depression, autoimmune diseases, and breast cancer treatment (35).	Sex hormones and chromosomes, immune modulation, epigenetic, and social factors likely contribute to differences in PAH prevalence and outcomes (40).	Shared mechanisms include heightened immune activation, interferon signaling, and estrogen–immune crosstalk. Autoimmunity prevalence is ∼2–3× higher in women and overlaps with PAH susceptibility (41, 42).
Anatomical/hemodynamic factors	Women have smaller coronary arteries and higher vessel curvature, which may influence shear stress and plaque formation differently than in men (43–45).	Generally, women maintain right ventricular (RV) adaptation despite higher PAH incidence. Males with PAH tend to have worse right ventricular (RV) function (40, 46).	Sex differences in ventricular–vascular coupling, mitochondrial efficiency, and oxidative stress handling favor RV adaptation in females. Male PAH patients show a higher risk of RV failure and death (47–49).
Hormonal or Estrogen influence	Postmenopausal, loss of estrogen raises LDL, lowers HDL, and accelerates atherosclerosis. Pre-menopausal women benefit from estrogen: improved lipid profiles, endothelial function, and metabolic regulation (50–52).	Estrogen contributes to the “sex paradox”; women are more likely to develop PAH yet often have better right ventricular function and survival (40, 46, 53).	Estrogen signaling can promote pulmonary vascular remodeling while protecting RV function; estradiol improves pulmonary arterial compliance/stiffness and RV adaptation (54–56).
Summary of disparity	Women's risk for ASCVD is lower before menopause, but certain risk factors (e.g., smoking, diabetes) hit women harder; postmenopausal risk accelerates, and unique factors contribute disproportionately (31).	Women are more likely to develop PAH but tend to survive longer with better RV function; risk factors manifest differently between sexes, and mechanisms are still under active investigation (40).	Divergent effects of sex hormones on systemic vs. pulmonary vasculature, coupled with sex-specific immune and metabolic programming, drive opposing risk and outcome profiles across ASCVD and PAH.

CAD; coronary artery disease, CVD; cardiovascular disease, LDL; low-density lipoprotein, HDL; high-density lipoprotein, BMI; body mass index, PASP; pulmonary artery systolic pressure.

This article aims to summarize the similarities between ASCVD and PAH regarding sex as a key biological factor, and to address the significant knowledge gap between preclinical animal studies and clinical data related to the two diseases, especially concerning sex disparities. Gaining a more comprehensive understanding of these mechanisms will not only clarify sex disparities but also help develop targeted therapeutic and preventive strategies, ultimately advancing personalized medicine for both women's and men's cardiovascular health.

## 2 ASCVD vs. PAH: the role of sex hormones

Both ASCVD and PAH exhibit apparent sex differences in both risk and disease development (54, 55). Past studies have focused on sex hormones such as estrogen and testosterone and their metabolites, vascular signaling pathways (e.g., BMPR2), inflammation, metabolic changes, RV alterations, and collider-stratification bias to explain these sex-based discrepancies.

**Figure 2:**
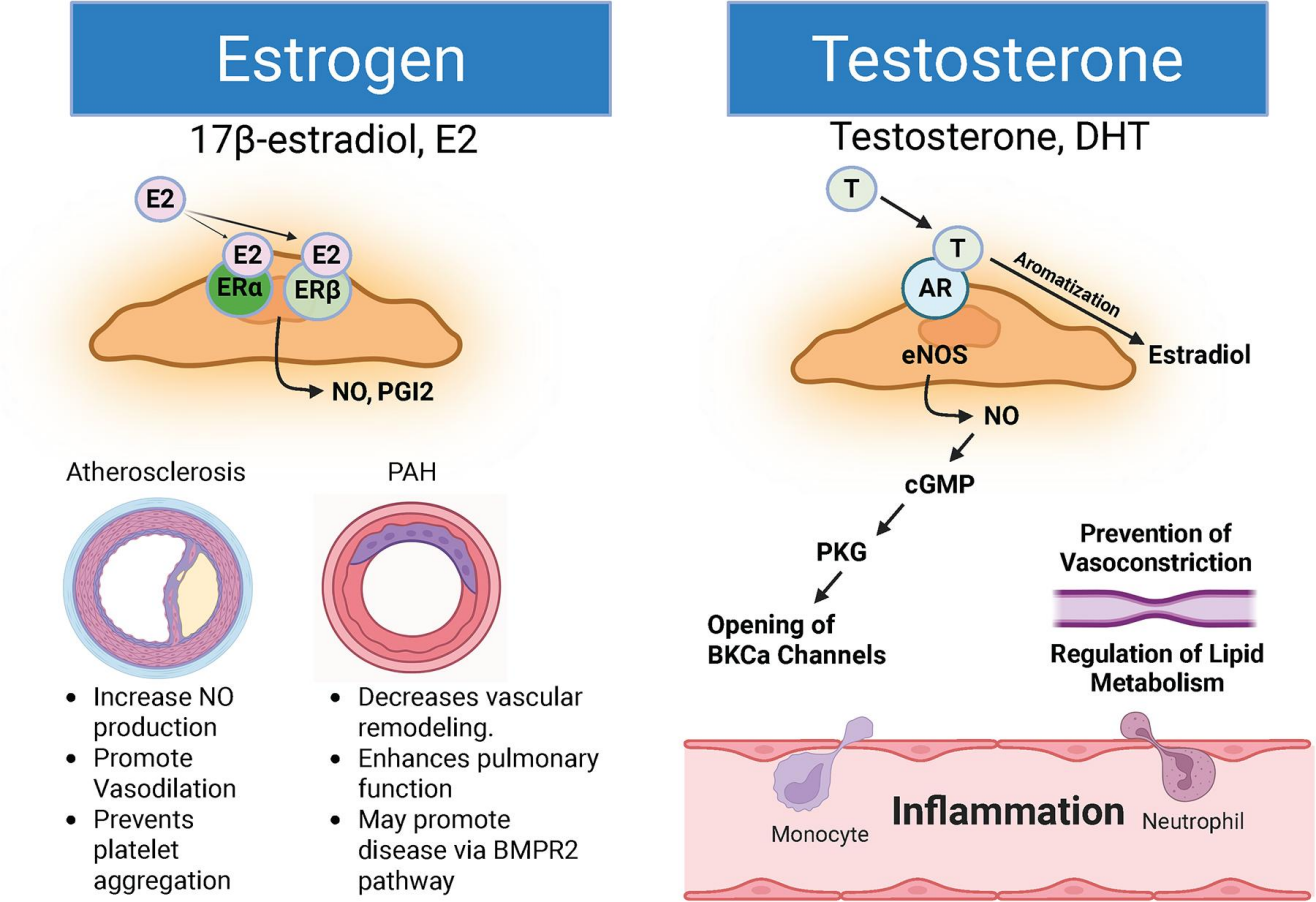
The schematic diagram illustrates the molecular mechanisms underlying estrogen (E2, or 17*β*-estradiol) and testosterone. Estrogen or 17*β*-estradiol (E2) is recognized for its ability to bind to ER*α* or ER*β*, thereby promoting the production of nitric oxide (NO) or prostaglandins (PGI2). In the context of atherosclerosis, the binding of E2 to ER enhances NO synthesis, facilitates vasodilation, and inhibits platelet aggregation; collectively, these functions constitute E2's atheroprotective role. Conversely, in pulmonary arterial hypertension (PAH), E2/ER*α* interaction stimulates NO production. In contrast, E2/ER*β* interaction promotes prostaglandin synthesis, predominantly I2, which is associated with vasodilation, reduced pulmonary vascular remodeling, and improved pulmonary vascular function. Furthermore, E2 is implicated in promoting the BMPR2-PAH disease pathway. On the other hand, Testosterone and dihydrotestosterone (DHT) activate androgen receptor (AR) signaling in endothelial cells, promoting endothelial nitric oxide synthase (eNOS) activation and nitric oxide (NO) production. NO stimulates the cGMP–protein kinase G (PKG) pathway in vascular smooth muscle, leading to opening of large-conductance Ca^2^^+^-activated K^+^ (BKCa) channels and reduced vasoconstriction. Testosterone, but not DHT, may also be aromatized to estradiol via aromatase, enabling estrogen receptor–dependent signaling that contributes to improved vascular function, lipid metabolism, and right ventricular adaptation. Its specific role in PAH remains inadequately defined. *Generated in biorender.

Estrogen exerts well-studied protective effects on the vasculature in both systemic atherosclerosis and pulmonary vascular disease (Figure 2). Activation of estrogen receptor-alpha (ER*α*) by estradiol (E2) increases endothelial NO production, accelerates re-endothelialization, and prevents vasoconstriction, making ER*α* signaling a key anti-atherogenic mechanism (57). Loss-of-function mutations in ESR1, the gene encoding ERɑ, have been linked to endothelial cell dysfunction, myocardial infarction, coronary artery disease, and stroke, highlighting estrogen's protective effects against atherosclerosis (57).

### 2.1 The estrogen paradox in PAH: mechanisms, metabolites, and unresolved questions

PAH demonstrates a well-known estrogen paradox, where women are more often affected but surprisingly have better survival rates compared to men. Registry data consistently show a higher prevalence in females in PAH cohorts and better survival outcomes (Table 1), but traditional risk profiles do not fully explain these sex differences, prompting further mechanistic research (55). Mechanistic insights implicate estrogen signaling, receptor biology, and metabolic pathways in modulating pulmonary vascular and RV responses. Activation of both estrogen receptors ERɑ and ER*β* in cultured pulmonary artery endothelial cells (PAECs) increases production of endothelial nitric oxide synthase (eNOS) and causes vasodilation (58). Additionally, ER*β*-mediated signaling is specifically associated with increased prostacyclin production, a NO-independent mechanism that promotes systemic vasodilation and protects against platelet aggregation (59). In PAH, estradiol can decrease pulmonary arterial muscularization, reduce lung inflammation and fibrosis, and promote pulmonary neoangiogenesis, thereby improving pulmonary function in women (54). Moreover, animal studies demonstrate that ovariectomy worsens PAH phenotypes, while estrogen repletion improves pulmonary vascular remodeling and enhances RV adaptation (60), supporting a protective role for estrogen in cardiopulmonary health. However, other studies suggest that estradiol can also aggravate PAH by modulating the bone morphogenetic protein receptor type II (BMPR2) pathway (61). Loss-of-function mutations in BMPR2 are the most common genetic cause of heritable PAH and are associated with a worse prognosis in both males and females (62). Additionally, sex differences in RV function and adaptation are increasingly recognized as central to the paradox; women tend to maintain better RV contractility and coupling under increased afterload, which correlates with improved survival outcomes (53–55). Multiple non-exclusive mechanisms have been proposed below for the “estrogen paradox in PAH”:

#### 2.1.1 Divergent roles in estrogen metabolites

Emerging evidence suggests that estrogen metabolites, rather than estradiol (E2) itself, may be the main drivers of different pulmonary vascular effects. 16*α*-hydroxyestrone (16*α*-OHE1) is produced by CYP1B1, an enzyme that is highly expressed in pulmonary artery lesions in patients with PAH and in cell cultures. *In vitro*, 16*α*-OHE1 promotes cell proliferation, enhances pulmonary arterial smooth muscle cell (PASMC) growth, and worsens pulmonary vascular remodeling; it is elevated in carriers of BMPR2 mutations (63). Conversely, 2-methoxyestradiol (2-ME2) is another estrogen metabolite that exhibits antiproliferative effects, blocks hypoxia-inducible factor-1*α* (HIF-1*α*), inhibits PASMC proliferation, and promotes apoptosis and microtubule destabilization (63, 64). A shift toward favoring 16*α*-OHE1 may increase the risk of PAH, while higher levels of 2-ME2 could offer protective effects against disease progression. This metabolite-specific duality is a key aspect of the estrogen paradox.

#### 2.1.2 ERɑ vs. ER*β* vs. GPER1: receptor subtype distribution matters

Estrogen receptors are not uniformly distributed across the systemic and pulmonary vasculatures. ERɑ is abundant in systemic endothelium and is strongly atheroprotective via NO signaling (57–59). ER*β* is enriched in the pulmonary vasculature and promotes prostacyclin synthesis, but may also enhance proliferative responses in BMPR2-deficient states (59). Lastly, GPER1 (G-protein-coupled estrogen receptor), a membrane-associated estrogen receptor independent of ER*α* and ER*β* mediates rapid non-genomic vasodilatory signaling and is upregulated during vascular stress (65). Disproportionate ER*β* and GPER1 activation in the pulmonary circulation may help explain why estrogen can simultaneously promote vasodilation while facilitating maladaptive remodeling (66, 67).

Similar to its role in cardiovascular aging, estrogen may exert protective effects early in pulmonary vascular disease but promote pathology in later stages of life. In the early years, patients may generally have a healthy endothelium; therefore, estrogen may promote anti-inflammatory effects, vasodilation, and antiproliferative effects. However, in patients with established injury due to aging or disease, estrogen may promote PASMC proliferation, inflammation, and remodeling, especially in BMPR-2-deficient environments (68). This hypothesis offers a potential temporal dimension to the estrogen paradox and warrants further investigation.

### 2.2 Testosterone and its metabolites in ASCVD and PAH

Testosterone and its metabolite dihydrotestosterone (DHT) influence vascular function in both ASCVD and PAH (Figure 2), although their roles remain less clearly defined. Beyond serving as a precursor for estradiol, testosterone exerts direct vascular effects through androgen receptor (AR) signaling in endothelial and smooth muscle cells (69). Physiological testosterone levels promote NO production, suppress vascular inflammation, and improve lipid metabolism, whereas testosterone deficiency is associated with increased ASCVD risk, metabolic syndrome, and endothelial dysfunction (70–73). Of note, in postmenopausal women, DHT levels decline to near-undetectable levels, which may partially explain the steep rise in atherosclerotic disease risk after age 50 (74). In PAH, high- but not low testosterone levels in men correlate with worse RV function and reduced survival, suggesting that testosterone may have a detrimental rather than protective effect in RV adaptation (75). Moreover, in experimental PAH studies, testosterone has been shown to mediate pulmonary vascular remodeling and vasodilation; however, this effect is limited to isolated vessels (76). Because *in vivo* studies of testosterone confirmed its detrimental effects on RV outcomes, it does not consistently offer complete protection against disease progression. Furthermore, due to limited data availability, its impact on pulmonary vascular remodeling, metabolism, and PASMC proliferation warrants further study.

Clinical insights into androgen signaling are further provided by studies of androgen deprivation therapy (ADT) in prostate cancer. ADT is associated with increased risk of myocardial infarction, stroke, heart failure, and metabolic dysfunction, highlighting the cardioprotective role of androgens (77, 78). These findings reinforce the importance of balanced androgen signaling in vascular health and suggest that excessive suppression of androgen pathways may have adverse cardiovascular consequences (79). Because testosterone serves as a precursor for estradiol via aromatization, it contributes to age-related hormonal changes affecting ASCVD risk in women. Notably, aromatase (estrogen synthase) is expressed in vascular endothelial cells, smooth muscle cells, and cardiomyocytes, enabling local conversion of testosterone to E2 within the vessel wall (Figure 2). This local production is believed to contribute to cardiovascular health by mediating the protective effects of E2 (80). By contrast, in PAH, evidence supports increased aromatase activity in pulmonary vascular lesions, which may amplify estrogen signaling independently of circulating hormone levels, thereby contributing to sex-specific vascular remodeling (81). This local estrogen production may partially explain why hormonal effects persist even in postmenopausal women and aging men. Given that estrogen metabolites, rather than estradiol alone, appear central to PAH pathobiology, clarifying the interplay between testosterone, AR signaling, and downstream conversion to E2 and DHT remains an important future direction.

## 3 Sex chromosomes as modulators in ASCVD and PAH

**Figure 3:**
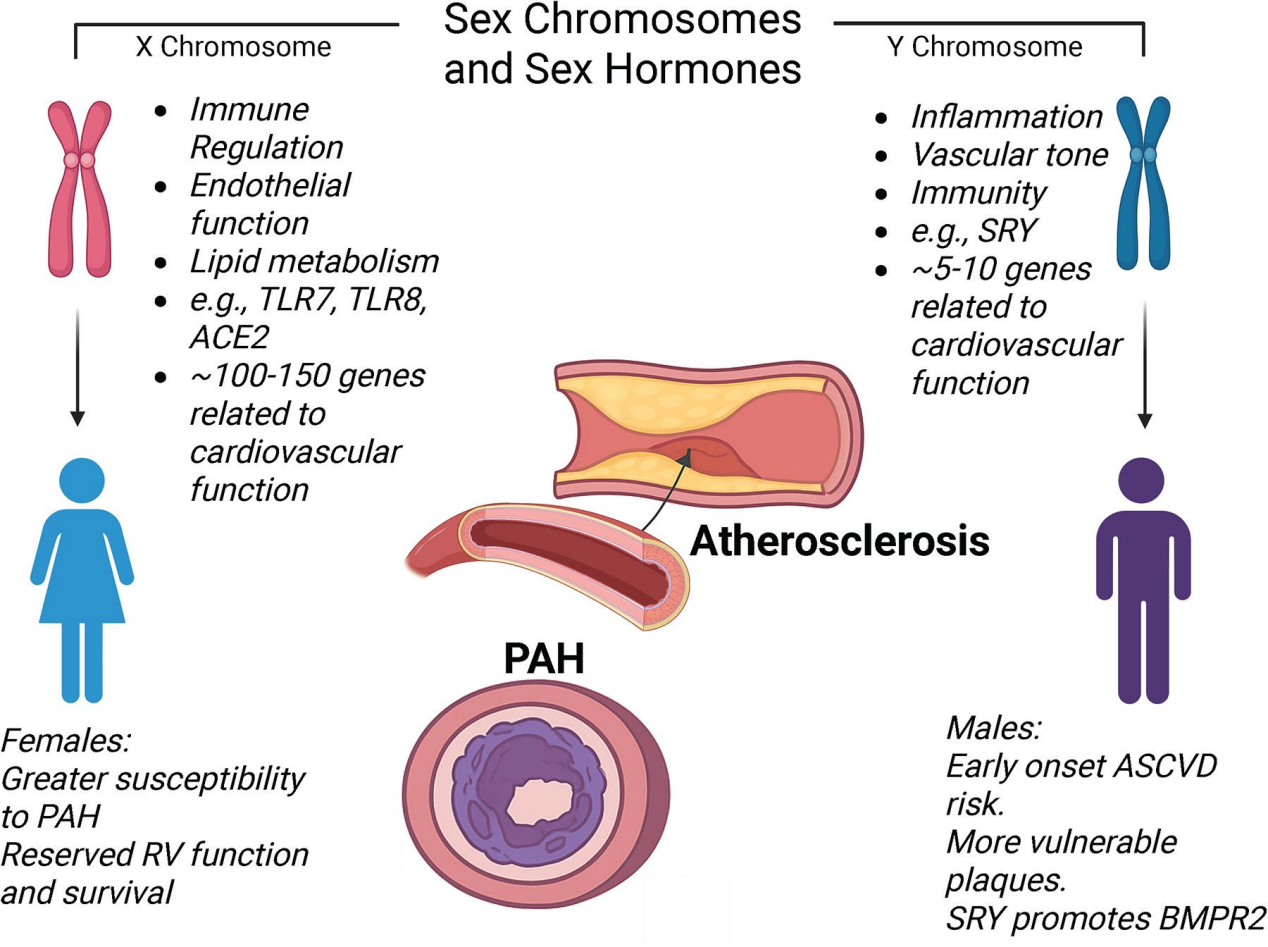
Schematic illustration of sex chromosome and hormone differences in relation to ASCVD and PAH. A list of genes on the X and Y chromosomes relevant to cardiovascular function is illustrated. *Generated in biorender.

Sex chromosomes (XX and XY) play pivotal roles in influencing susceptibility to vascular diseases, including ASCVD and PAH (Figure 3). The X chromosome encompasses numerous genes involved in immune regulation, endothelial function, and lipid metabolism (82–84). Females possess two X chromosomes, which may confer their resilience in vascular adaptation via gene-dosage effects or X-linked gene escape from inactivation (85, 86). Importantly, several X-linked genes that escape X-inactivation are expressed at higher levels in females, including KDM6A and DDX3X (87, 88). KDM6A (lysine demethylase 6A) and DDX3X (DEAD-box RNA helicase X-linked) are involved in inflammation and vascular biology that may intersect with atherosclerotic and PAH processes. Human studies of X chromosome escape genes show KDM6A (UTX) escapes inactivation and is active in cardiometabolic traits, including lipid metabolism and CVD risk profiles (89). Moreover, experimental studies suggest that KDM6A regulates inflammatory gene expression, including pro-inflammatory cytokines (e.g., IL-6, IFN-*β*) via H3K27 demethylation in innate immune cells such as macrophages, a key process linked to vascular inflammation and atherosclerosis pathogenesis (90). However, the direct effect of KDM6A on atherosclerosis remains largely uninvestigated. In contrast, DDX3X encodes an ATP-dependent RNA helicase that is critically involved in RNA metabolism, including translation initiation and RNA spliceosome assembly, and also serves as a node in innate immune and stress-responsive pathways (89). Although direct studies of atherosclerosis are limited, DDX3X has been implicated in innate immune responses, including inflammasome activation and NF-*κ*B signaling, which are central to atherogenic endothelial dysfunction and macrophage activation in vascular lesions (91), making it a plausible contributor to chronic vascular inflammation in atherosclerosis. However, how these gene escape effects influence overall female ASCVD risk and management is largely unknown.

In PAH, increased expression of these genes may enhance immune responsiveness and vascular adaptability in females, contributing to higher PAH susceptibility while improving RV resilience (87). BMPR2 loss-of-function mutations create a permissive genetic background for PAH, but disease penetrance remains incomplete, suggesting that other modifiers are needed for clinical development. Among BMPR2 mutation carriers, penetrance shows a significant sex bias, with females developing PAH much more often than males (∼40% vs. 14%–15%, respectively), despite similar rates of mutation carriage (92, 93). In males, the SRY (sex-determining region Y) gene has been shown to influence transcriptional programs beyond sex determination, including aiding BMPR2 expression, which supports pulmonary vascular stability and may reduce disease penetrance (94). However, PAH risk is not solely dictated by BMPR2 expression. In females, estrogen signaling and metabolism act downstream of BMPR2 to promote pulmonary vascular cell growth, inflammation, and resistance to cell death, thereby increasing disease risk even when BMPR2 signaling remains intact (95, 96). These findings support a “two-hit” model of PAH development, in which a BMPR2 mutation is the first hit. A second hit, such as sex hormone signaling, inflammation, hypoxia, metabolic stress, pregnancy, or environmental exposures, is necessary to induce overt pulmonary vascular remodeling and clinical symptoms (97). Consistent with this idea, unaffected BMPR2 mutation carriers display higher adequate BMPR2 transcript levels than those with the disease, highlighting the significance of both genetic background and modifying factors in determining disease penetrance (98).

The interaction between sex chromosomes and sex hormones further modulates disease outcomes. For instance, X-linked immune genes, such as toll-like receptors TLR7 and TLR8, can amplify inflammatory pathways that facilitate ASVD and PAH, whereas estrogen confers protective effects (99, 100). These chromosomal effects underscore that sex differences in vascular disease are not solely hormone-dependent but are also genetically encoded, influencing how vessels respond to stress, inflammation, and remodeling in both the systemic arterial and pulmonary circulations*.* Insights derived from the Four Core Genotypes (FCG) mouse model, which separates sex chromosome complement from gonadal sex by relocating the SRY gene to an autosome, resulting in XX and XY mice with either testes or ovaries (101). This model facilitates the identification of sex-chromosome-dependent phenotypes independent of circulating hormones and has been employed to explore sex-chromosome influences on cardiovascular and pulmonary phenotypes, including ischemia/reperfusion injury and pulmonary hypertension models (102). Additionally, FCG studies underscore that variations in X chromosome escape genes may affect vascular responses, suggesting that chromosomal components beyond traditional hormonal mechanisms are involved (101–103). However, sex chromosomal genes receive little attention, even though various functional studies on cardiovascular health have been conducted in both animal models and humans; therefore, future studies should further investigate the role of sex-linked genes in atherosclerosis and PAH.

## 4 Emerging sex-specific modulators linking ASCVD and PAH

### 4.1 MicroRNAs: sex-specific post-transcriptional regulation

MicroRNAs (miRNAs) are increasingly recognized as critical regulators of vascular homeostasis and disease progression, with accumulating evidence that their expression and function are sex dependent. In both ASCVD and PAH, dysregulated miRNA profiles contribute to endothelial dysfunction, smooth muscle cell proliferation, inflammation, and adverse cardiac remodeling (104). Among these, miR-29, miR-124, and miR-204 have emerged as key sex-sensitive regulators across systemic and pulmonary vasculature. MiR-29 regulates extracellular matrix turnover and fibrosis; its downregulation promotes vascular stiffening and remodeling in ASCVD and PAH, with evidence of estrogen-sensitive regulation (104). MiR-124 suppresses inflammatory and proliferative signaling in PASMCs and is reduced in PAH, particularly in female-derived cells (105). MiR-204 is consistently downregulated in PAH and contributes to STAT3 activation, inflammation, and vascular remodeling; estrogen-mediated suppression of miR-204 provides a mechanistic link between sex hormones and PAH susceptibility (105, 106). Collectively, these miRNAs represent shared, sex-modulated regulators across systemic and pulmonary vascular disease.

### 4.2 Metabolomics: sex differences in energy utilization

Sex differences in metabolic substrate utilization significantly influence vascular disease progression and cardiac adaptation. In ASCVD, men exhibit more adverse lipid profiles and impaired fatty acid oxidation, whereas premenopausal women demonstrate greater metabolic flexibility, an advantage lost after estrogen decline in menopause (107). In PAH, pulmonary vascular cells and the RV undergo metabolic reprogramming toward glycolysis. Female RV cells maintain more efficient mitochondrial oxidative metabolism and fatty acid utilization, contributing to superior RV adaptation and survival compared to males (47, 108). These sex-specific metabolic phenotypes highlight metabolomics as a critical determinant of disease severity and outcomes.

### 4.3 Gut microbiome: an emerging sex modifier

Emerging evidence implicates the gut microbiome as a sex-dependent modulator of cardiovascular disease. In ASCVD, microbiota-derived metabolites such as trimethylamine N-oxide (TMAO) are higher in men and correlate with increased atherosclerotic risk, whereas estrogen suppresses TMA-producing microbial pathways (109, 110). Although data on the gut microbiome in PAH are limited, altered microbial diversity has been linked to systemic inflammation and metabolic dysfunction, suggesting that sex-dependent microbiome-immune interactions may contribute to pulmonary vascular remodeling and RV dysfunction (111).

### 4.4 Epigenetics: hormone-responsive gene regulation

Epigenetic mechanisms, such as DNA methylation and histone modifications, mediate long-term effects of sex hormones on vascular gene expression. Estrogen receptor signaling promotes histone acetylation and transcription of vasoprotective genes, while estrogen loss is associated with pro-atherogenic epigenetic changes (112). In PAH, epigenetic repression of BMPR2 and anti-proliferative pathways contributes to disease progression, with sex hormones influencing these epigenetic markers and potentially explaining sex-specific penetrance in mutation carriers (113).

### 4.5 Mitochondrial function and RV adaptation

Sex differences in mitochondrial function critically influence RV adaptation in PAH. Female RV mitochondria exhibit enhanced oxidative capacity, improved antioxidant defenses, and resistance to oxidative stress, largely mediated by estrogen-dependent pathways (114, 115). In contrast, male RVs demonstrate greater mitochondrial dysfunction and oxidative injury under pressure overload, contributing to worse survival rates (115). These findings underscore mitochondrial metabolism as a sex-specific determinant of PAH and outcomes.

## 5 Lesson learned from rodent models for sex differences

The underrepresentation of women in clinical trials and genomic datasets contributes significantly to the persistent disparities in understanding sex differences in ASCVD and PAH. To enhance understanding of these sex-related differences in ASCVD and PAH, *in vivo* rodent models are valuable tools that offer insights into biological distinctions and mechanisms attributable to sex. Table 3 summarizes rodent models of PAH and atherosclerosis, including a comparison of sex differences in their development. In atherosclerosis research, both diet-induced approaches and genetically modified strains, such as apolipoprotein E-deficient (ApoE^−^/^−^) and low-density lipoprotein receptor–deficient (LDLR^−^/^−^) mice, have been widely used to study lipid imbalance and plaque formation (116, 117). In PAH, experimental models such as chronic hypoxia (118), monocrotaline (MCT) treatment (119), and the Sugen–hypoxia models (120) reproduce the hallmark features of pulmonary vascular remodeling, endothelial dysfunction, and RV adaptation observed in human idiopathic PAH. Table 3 summarizes rodent models that may be used to investigate ASCVD and PAH simultaneously, including a comparison of sex differences. PAH animal models are fully reviewed (121), and the ASCVD rodent model is thoroughly reviewed (122).

**Table 3:** Rodent Models of ASCVD and PAH: Insights into Sex-Specific Mechanisms.

Animal Model	Disease Model	Sex-Differences Findings	Overlap (PAH & ASCVD in the same model?)	References
ApoE^−^/^−^ mouse	Atherosclerosis	Females: Spontaneously form smaller, more fibrotic plaques; protection is lost after estrogen decline with age. Males: larger, lipid-rich plaques.	Yes, used in ASCVD & PAH studies. Mechanism, including inflammation & vascular remodeling.	(7, 123, 124).
LDLR^−^/^−^ mouse	Atherosclerosis	Females: delayed lesion progression and more calcification with the Western diet Males: more severe hyperlipidemia and plaque burden.	Primarily the ASCVD model, but occasionally used in pulmonary vascular remodeling under dietary stress.	(125)
CRISPR ApoE^−^/^−^/LDLR^−^/^−^ rat (single/double KO)	Atherosclerosis	Females: milder lesions. Males: extensive plaque burden and dyslipidemia.	No established ASCVD and PAH overlap.	(126)
Chronic Hypoxia (mouse/rat)	PAH	Females are more likely to develop PAH but often maintain better RV function than males.	Does not induce atherosclerosis.	(114)
Monocrotaline (MCT) rat	PAH	Females: better RV adaptation; ovariectomy worsens outcomes, estrogen restores function. Males: worse RV function and survival due to oxidative stress.	No, liver metabolism of MCT is specific to PAH.	(65, 127)
Sugen–Hypoxia (SuHx) rat/mouse	PAH	Females: severe pulmonary vascular remodeling but preserved RV function; estrogen supplementation improves outcomes. Males: severe pulmonary vascular remodeling and RV function deteriorate.	No, primarily PAH.	(60, 115, 128)
Zucker Obese rat	PAH (MCT-induced) & Metabolic syndrome	Female obese rats: more severe PAH vs. lean females; sex differences are less marked in hypoxia models.	This model partially reflects cardiometabolic risk relevant to both ASCVD and PAH.	(129)
BMPR2 mutant mouse/rat	PAH	Females: predisposition to PAH; hormonal modulation alters penetrance. Males: no spontaneous development of PAH.	ASCVD is not typically studied.	(95, 130, 131)

It is noteworthy that animal models often do not fully replicate the human ASCVD and PAH phenotypes, including their structural and hemodynamic intricacies, species-specific cardiopulmonary responses, and the impact of sex hormones on disease manifestation. Most rodent models rely on single insults, such as dietary, toxic, hypoxic, or genetic insults, and do not capture the chronic, multifactorial nature of human disease. In ASCVD, fundamental species differences in lipoprotein metabolism limit modeling of plaque instability and clinical events, whereas in PAH, key histopathological features, such as plexiform lesions and progressive RV failure, are inconsistently reproduced. Moreover, experimental manipulation of sex hormones in rodents (e.g., ovariectomy or castration) oversimplifies human hormonal transitions such as menopause and androgen decline, potentially exaggerating sex effects or misrepresenting disease biology (53, 132). Age is an essential limitation across most ASCVD and PAH animal models, as experiments are typically performed in young animals. In contrast, clinical disease most often manifests in middle-aged or older individuals. Aging profoundly alters vascular biology, including endothelial dysfunction, mitochondrial impairment, immune senescence, and extracellular matrix remodeling, which can modify disease penetrance, severity, and therapeutic responses, limiting direct translation from young-animal studies to older patient populations (133–135).

Species-specific differences further influence susceptibility to PAH and interpretation of sex effects. Rats generally develop more severe pulmonary hypertension with robust pulmonary vascular remodeling and RV hypertrophy, making them advantageous for studying advanced disease and RV adaptation. In contrast, mice exhibit strain-dependent susceptibility, milder hemodynamic changes, and limited progression to RV failure despite their utility for genetic manipulation (121). Sex differences are also model- and species-dependent: female rats consistently demonstrate superior RV adaptation in MCT and Sugen–hypoxia models, whereas sex-related differences are minimal or absent in chronic hypoxia–induced PAH in mice, highlighting divergence between vascular pathology and cardiac adaptation across species (114, 129). These differences underscore the importance of selecting species and models based on the biological question rather than the disease label alone.

Emerging human-relevant model systems offer complementary approaches to address these translational gaps. Human-induced pluripotent stem cell-derived endothelial and smooth muscle cells, lung and vascular organoids, and precision-cut lung slices enable interrogation of sex-specific vascular, metabolic, and immune mechanisms within a human genetic and hormonal context (136–138). In addition, humanized mouse models incorporating patient-derived cells or immune components may better capture inflammatory and sex-dependent features (139) relevant to both ASCVD and PAH. Although these systems cannot yet model long-term hemodynamics or systemic interactions, they provide critical platforms for mechanistic validation and hypothesis refinement before *in vivo* testing.

Accordingly, optimal model selection for guided testing sex-specific hypotheses should be driven by the experimental intent. Genetic mouse models are best suited for pathway-specific and immune-mediated mechanisms, rat models for severe pulmonary vascular remodeling and RV adaptation, and humanized or *ex vivo* systems for investigating sex hormone signaling and cell-intrinsic differences relevant to PAH–ASCVD overlap. Integrating findings across complementary models rather than relying on a single system will be essential for improving translational relevance and advancing sex-informed therapeutic strategies.

## 6 Clinical implications

**Figure 4:**
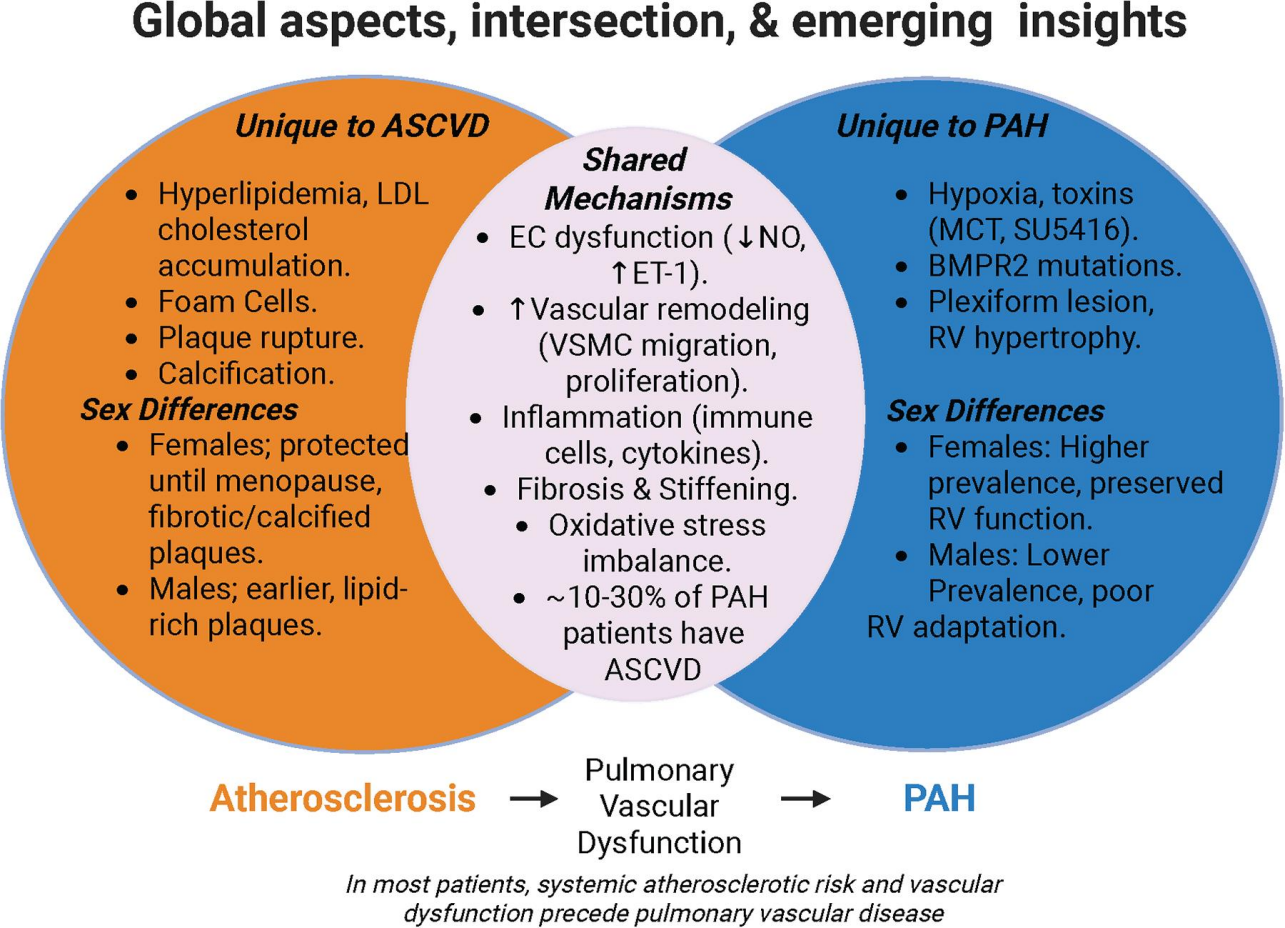
Schematic showing the unique and shared mechanisms between ASCVD and PAH. *Generated in biorender.

Although ASCVD and PAH arise from distinct initial causes, they share remarkably similar pathophysiological mechanisms (140, 141) (Figure 4). In fact, advanced atherosclerotic lesions have been observed in patients with long-standing or advanced PAH (142), highlighting the possible overlap in disease mechanism between these vascular disorders (99, 143). Mechanistically, several atherosclerotic pathways intersect with PAH pathobiology, including arterial wall remodeling, endothelial dysfunction, smooth muscle cell proliferation, inflammation, and fibrosis (140, 141). These common processes suggest that the coexistence of ASCVD and PAH in the same patient is not coincidental but may reflect shared underlying drivers of vascular injury and maladaptation. Significantly, sex further influences these pathways. Female patients with ASCVD and PAH often experience greater disease severity and different progression patterns compared to males (44, 144), implicating hormonal, immune, and molecular mechanisms in shaping outcomes. Therefore, it is both essential and timely to investigate sex-specific mechanisms at the intersection of ASCVD and PAH, as sex differences extend beyond prevalence and encompass variations in pathophysiology, circulating biomarkers, and treatment responses in these two conditions diseases.

### 6.1 Early clinical screening for ASCVD and PAH, along with sex stratification

Sex should be explicitly included in clinical risk stratification algorithms for both ASCVD and PAH. In PAH, existing tools like REVEAL and COMPERA already factor in sex, but growing evidence indicates that more detailed stratification is necessary based on other sex-specific factors, including menopausal status, cardiometabolic burden, and RV adaptive capacity (15, 16, 33, 144). Conversely, ASCVD appears earlier in men but progresses more quickly in postmenopausal women after the loss of estrogen-related vascular protection (1). Therefore, postmenopausal women with PAH and men with PAH who have metabolic risks should receive routine ASCVD screening. Likewise, female patients with ASCVD who present with unexplained dyspnea or RV dysfunction should be evaluated for PAH. These points support a sex-informed, bidirectional risk assessment approach across both conditions.

Recognition of sex differences in circulating cardiovascular biomarkers has significant implications for the early detection and risk stratification of both ASCVD and PAH. Women generally exhibit higher circulating levels of natriuretic peptides, such as B-type natriuretic peptide (BNP) and N-terminal pro B-type natriuretic peptide (NT-proBNP), than men of comparable age and hemodynamic status (145, 146). This phenomenon has been documented across multiple populations (147) and can influence the interpretation of cardiac stress markers. Similarly, concentrations of high-sensitivity cardiac troponin (hs-cTn) vary by sex, with men typically having higher baseline values and women exhibiting lower 99th-percentile reference limits (148), necessitating sex-specific thresholds in diagnostic algorithms. These differences mirror physiological variations in cardiac structure, metabolism, and hormonal regulation, and are not limited to heart failure but extend to a broader spectrum of cardiovascular conditions. Recognizing these sex-specific biomarker distributions holds clinical significance; the application of uniform cutpoints may delay diagnosis or result in underrecognition of myocardial injury and ventricular dysfunction in women. Conversely, sex-adjusted thresholds have the potential to enhance diagnostic sensitivity, prognostication, and equitable clinical care in both ASCVD and PAH without compromising specificity.

In addition to circulating biomarkers, advances in cardiopulmonary imaging are enhancing the detection of vascular and RV dysfunction in individuals at risk for, or diagnosed with, ASCVD and PAH. Non-invasive echocardiographic markers such as pulmonary artery acceleration time (PAcT), RV–pulmonary artery (RV-PA) coupling indices, and RV longitudinal strain provide surrogate measures of pulmonary vascular load and RV performance and may detect subclinical dysfunction before the development of overt pulmonary hypertension or symptomatic RV failure (149, 150). Significantly, emerging evidence indicates sex-related differences in RV adaptation and imaging phenotypes: female patients with PAH exhibit greater RV contractility and RV-PA coupling than their male counterparts, despite comparable afterload (47). This variability may help explain the observed sex disparities in clinical outcomes and can be identified through imaging evaluations, such as RV strain and RV–PA coupling measures. Cardiac magnetic resonance imaging (CMR) offers highly reproducible measurements of RV volumes, mass, and functional adaptation, establishing itself as the benchmark for detecting subtle myocardial remodeling. Population-based research indicates sex-dependent differences in RV strain and function in CMR (151), and these distinctions should inform clinical assessment and interpretation.

Furthermore, CT-based assessments, which include quantitative measures of pulmonary vascular pruning and the concurrent evaluation of coronary artery calcium, enable the comprehensive characterization of pulmonary and systemic vascular disease burdens within a single imaging session (152). Notably, quantitative CT measures of pulmonary vascular pruning have been shown to reflect the loss of small pulmonary vessels, to serve as an imaging surrogate for pulmonary vascular disease, and to have prognostic value for mortality and cardiopulmonary outcomes in PAH (152). Coronary artery calcium scoring on CT is a well-established marker of systemic atherosclerotic burden and cardiovascular risk (153). These imaging biomarkers can often be derived from the same or similar chest CT data, enabling simultaneous assessment of both pulmonary and systemic vascular disease. While these advanced imaging techniques provide additional diagnostic and prognostic information beyond biomarkers alone, routine population-wide screening for ASCVD and PAH using high-resolution imaging is unlikely to be cost-effective. Targeted screening strategies that incorporate clinical risk factors, including biological sex and metabolic risk, biomarker profiles, and sex-informed non-invasive imaging in higher-risk groups are more practical and may support earlier diagnosis and treatment, aligning with current guidelines that emphasize risk-based rather than universal screening.

### 6.2 ASCVD and PAH overlapping management and sex significance

Sex-specific differences in therapeutic responses have become a significant consideration in managing ASCVD and PAH, and emphasize the translational importance of sex as a biological variable (12, 13, 154, 155). Clinical evidence indicates that although both women and men derive substantial benefits from standard lipid-lowering therapies, notable disparities exist in response magnitude and treatment patterns in ASCVD patients. For example, while statins effectively reduce low-density lipoprotein cholesterol (LDL-C) and major adverse cardiovascular events (MACE) in both sexes, women are generally less likely to be prescribed high-intensity statin therapy and to achieve guideline-recommended LDL-C targets than men (156–158). Observational and registry data for PCSK9 inhibitors show that, although these agents reduce LDL-C and cardiovascular events across sexes, sex differences in LDL-C reduction have been reported, with men sometimes achieving greater percentage reductions and women less frequently reaching lipid goals despite similar treatment indication (159, 160). Additionally, disparities in the initiation and intensification of therapies such as ezetimibe and PCSK9 inhibitors following myocardial infarction have been observed by sex (161), suggesting differences in clinical practice and response.

Observational and registry data in PAH suggest that female patients often exhibit better functional and hemodynamic responses to endothelin receptor antagonists and may experience more favorable RV adaptation and survival than male patients. In contrast, male sex has been associated with poorer RV adaptation and outcomes in many cohorts. The influence of sex on response to phosphodiesterase-5 inhibitors and on pulmonary vascular resistance changes remains less clearly defined (131, 144, 162, 163). Prostacyclin therapies improve functional and hemodynamic outcomes in both women and men with PAH; however, observational and mechanistic studies suggest that sex-dependent differences in RV adaptation, and possibly mitochondrial and oxidative signaling, may influence variability in RV response and tolerability to these agents (40, 47). Building on these observations, Sex-informed therapeutic strategies are now entering clinical testing in PAH, including estrogen pathway modulation with aromatase inhibitors such as anastrozole**,** which has been shown to lower circulating estradiol and improve exercise capacity in PAH patients (164), and the clinical evaluation of estrogen receptor antagonists such as fulvestrant in postmenopausal women with PAH is underway (165).

While most therapies approved for PAH target the pulmonary vasculature, accumulating evidence suggests that certain agents used to manage PAH may also exert beneficial effects across a broader spectrum of cardiovascular diseases, including ASCVD. For instance, phosphodiesterase-5 inhibitors (such as sildenafil and tadalafil), which are standard treatments for PAH, have been associated in observational studies and clinical trials with improved systemic endothelial function, reduced inflammatory markers, and a decreased risk of myocardial infarction and heart failure (166), suggesting potential cardioprotective effects beyond the pulmonary circulation. Likewise, endothelin receptor antagonists (including bosentan and ambrisentan) have demonstrated anti-atherosclerotic properties and endothelial benefits in preclinical models and small-scale clinical studies, including those involving peripheral artery disease, where they enhanced vascular function and lowered the risk of adverse events (167). Although comprehensive randomized clinical trials specifically assessing PAH drugs within primary or secondary ASCVD populations remain limited, these translational insights support the premise that agents modulating the nitric oxide-cyclic guanosine monophosphate (NO-cGMP) and endothelin pathways may provide therapeutic benefits across a range of cardiopulmonary vascular diseases, emphasizing shared mechanisms in PAH and ASCVD and encouraging further clinical investigation.

As statins are the primary agents in ASCVD owing to their potent LDL-cholesterol-lowering and plaque-stabilizing properties, their role in PAH has been extensively studied with mixed and largely inconclusive outcomes (168). In animal models of PAH, statins demonstrate beneficial vascular effects: they enhance endothelial function, reduce pulmonary vascular remodeling and RV hypertrophy, and lower inflammatory mediators (168), providing a mechanistic basis for their potential benefits. However, in human studies, evidence has not consistently shown clear improvements in function or hemodynamics. Early randomized trials, such as the Simvastatin as a Treatment for Pulmonary Hypertension study, observed reductions in RV mass and neurohormonal biomarkers (e.g., NT-proBNP) over short periods but no long-term benefits on exercise capacity, pulmonary pressures, or clinical outcomes (169). Systematic reviews and meta-analyses of available randomized and observational studies indicate that statins do not significantly improve key clinical endpoints in PAH, including the 6-minute walk distance, pulmonary artery pressure, cardiac index, or pulmonary vascular resistance (170). The disparity between animal models and human trials may be due to differences in disease mechanisms or the relative importance of lipid-independent (“pleiotropic”) effects of statins in pulmonary vs. systemic vascular tissues. Despite these mixed results, statin therapy is generally safe and well tolerated in PAH patients, with ongoing research focusing on better-powered or subtype-specific studies to determine if certain PAH phenotypes (or comorbid pulmonary hypertension groups) could benefit.

## 7 Conclusions

The relationship between atherosclerosis (ASCVD) and PAH remains incompletely understood. Advances in the molecular and pathophysiological characterization of both conditions should provide the foundation for multidisciplinary research efforts aimed at unraveling their interconnections. Such collaboration will not only clarify the clinical impact of comorbidity between ASCVD and PAH but also inform the development of novel therapeutic strategies and the prioritization of research funding to address these pressing gaps. While idiopathic PAH is a rare and challenging condition, frequently diagnosed using methods developed for ASCVD, both diseases share hallmark mechanisms, including chronic inflammation, immune dysregulation, smooth muscle cell abnormalities, endothelial dysfunction, and vascular remodeling. This convergence underscores the interconnected nature of systemic and pulmonary vascular diseases, highlighting the need for integrative approaches to study their overlapping biology and clinical manifestations. Importantly, sex-specific differences in the prevalence, severity, and outcomes of ASCVD and PAH are increasingly recognized at multiple levels, from hormonal regulation to gene expression and immune responses. These disparities not only shape disease development but also influence therapeutic responses. A deeper understanding of how sex hormones and sex-linked genetic factors influence vascular biology will be critical for advancing personalized prevention and treatment strategies. Ultimately, progress in this field will depend on recognizing sex as a central biological variable and on building research frameworks that incorporate sex-specific mechanisms into both experimental models and clinical practice. Future research must address the complexities of ASCVD and PAH by integrating sex-related genetic, molecular, and epigenomic approaches, alongside psychosocial and behavioral sciences, to thoroughly characterize sex-based disparities in vascular diseases. A coordinated research strategy is necessary, one that applies unified methodologies across ASCVD and PAH and leverages interdisciplinary collaborations among clinicians, basic scientists, and funding agencies to prioritize sex-based investigations. Moreover, several critical limitations shape the interpretation of the current evidence and point to opportunities for future research. Much of the reviewed literature originates from predominantly White, Western cohorts, and sex-by-race/ethnicity interactions remain insufficiently characterized, limiting the generalizability across diverse populations. In clinical studies, observed sex differences may also be confounded by age and hormonal status, especially in mixed pre- and post-menopausal cohorts, complicating the attribution of outcomes solely to biological sex. Publication bias is a potential concern, whereby studies reporting positive sex differences may be overrepresented relative to those reporting null findings. It is important to note that many proposed mechanisms, while supported by preclinical and observational studies, lack definitive validation in human cohorts. Although animal models are discussed as tools for mechanistic insight, these systems have inherent limitations in capturing the full complexity of human cardiopulmonary disease.

Furthermore, the focus on estrogen and testosterone, though central, oversimplifies the broader endocrine environment and does not fully account for interactions with other hormonal and metabolic pathways. Despite an expanding mechanistic literature, there has been limited translation into sex-specific therapies, underscoring the necessity for future studies that connect mechanistic understanding with clinical innovation and therapeutic development. Addressing disparities in trial enrollment, diagnosis, and treatment, as well as implementing sex-specific prevention and management strategies, will be essential to optimize outcomes for both women and men afflicted with ASCVD and PAH.

Future research examining these disorders through a sex-based perspective will be vital for uncovering causal pathways, improving risk assessment, and developing precision therapies tailored specifically for men and women, who carry a disproportionate burden of these vascular diseases.

## Glossary

ASCVD: Atherosclerotic cardiovascular disease
PAH: Pulmonary arterial hypertension
PH: Pulmonary hypertension
RV: Right ventricle / right ventricular
RVSP: Right ventricular systolic pressure
RV–PA coupling: Right ventricular–pulmonary artery coupling

## Hormones and receptors

E2: 17β-estradiol
ERα (ESR1): Estrogen receptor alpha
ERβ (ESR2): Estrogen receptor beta
GPER (GPER1): G protein–coupled estrogen receptor
AR: Androgen receptor
SRY: Sex-determining region Y

## Genetics and signaling

BMPR2: Bone morphogenetic protein receptor type II
BMP: Bone morphogenetic protein
eNOS: Endothelial nitric oxide synthase
NO: Nitric oxide

## Cell types

EC: Endothelial cell
PAEC: Pulmonary artery endothelial cell
PASMC: Pulmonary artery smooth muscle cell
VSMC: Vascular smooth muscle cell

## Animal models

ApoE^−^/^−^: Apolipoprotein E–deficient
LDLR^−^/^−^: Low-density lipoprotein receptor–deficient
MCT: Monocrotaline
SuHx: Sugen–hypoxia
ZSF1: Zucker diabetic fatty strain
OVX: Ovariectomy

## Metabolic and clinical terms

FH: Familial hypercholesterolemia
MI: Myocardial infarction
CAD: Coronary artery disease
BNP: B-type natriuretic peptide
NT-proBNP: N-terminal pro–B-type natriuretic peptide
hs-cTn: High-sensitivity cardiac troponin

## Imaging and diagnostics

CMR: Cardiac magnetic resonance imaging
CT: Computed tomography
PAAT: Pulmonary artery acceleration time

## Clinical trials and guidelines

EDIPHY: Effects of DHEA in Pulmonary Hypertension
ESC: European Society of Cardiology
ERS: European Respiratory Society

## References

[1] Martin SS Aday AW Allen NB Almarzooq ZI Anderson CAM Arora P 2025 Heart disease and stroke statistics: a report of US and global data from the American Heart Association. Circulation. (2025) 151(8):e41–e660. 10.1161/CIR.0000000000001303 39866113 PMC12256702

[2] Luna-López R Ruiz Martín A Escribano Subías P. Pulmonary arterial hypertension. Med Clin (Barc). (2022) 158(12):622–9. 10.1016/j.medcli.2022.01.003 35279313

[3] Ibanez B Fernández-Ortiz A Fernández-Friera L García-Lunar I Andrés V Fuster V. Progression of early subclinical atherosclerosis (pesa) study: jacc focus seminar 7/8. J Am Coll Cardiol. (2021) 78(2):156–79. 10.1016/j.jacc.2021.05.011 34238438

[4] Libby P. The changing landscape of atherosclerosis. Nature. (2021) 592(7855):524–33. 10.1038/s41586-021-03392-8 33883728

[5] Kidder E Gangopadhyay S Francis S Alfaidi M. How to release or not release, that is the question.” A review of interleukin-1 cellular release mechanisms in vascular inflammation. J Am Heart Assoc. (2024) 13(5):e032987. 10.1161/JAHA.123.032987 38390810 PMC10944040

[6] Alfaidi M Wilson H Daigneault M Burnett A Ridger V Chamberlain J Neutrophil elastase promotes interleukin-1beta secretion from human coronary endothelium. J Biol Chem. (2015) 290(40):24067–78. 10.1074/jbc.M115.659029 26269588 PMC4591798

[7] Renshall L Arnold N West L Braithwaite A Pickworth J Walker R Selective improvement of pulmonary arterial hypertension with a dual et(a)/et(B) receptors antagonist in the apolipoprotein E(-/-) model of pah and atherosclerosis. Pulm Circ. (2018) 8(1):2045893217752328. 10.1177/2045893217752328 29261014 PMC5798688

[8] Marinho Y Villarreal ES Loya O Oliveira SD. Mechanisms of lung endothelial cell injury and survival in pulmonary arterial hypertension. Am J Physiol Lung Cell Mol Physiol. (2024) 327(6):L972–l83. 10.1152/ajplung.00208.2024 39406383 PMC11684956

[9] Moore GW Smith RR Hutchins GM. Pulmonary artery atherosclerosis: correlation with systemic atherosclerosis and hypertensive pulmonary vascular disease. Arch Pathol Lab Med. (1982) 106(8):378–80. 6213213

[10] Mair KM Johansen AK Wright AF Wallace E MacLean MR. Pulmonary arterial hypertension: basis of sex differences in incidence and treatment response. Br J Pharmacol. (2014) 171(3):567–79. 10.1111/bph.12281 23802760 PMC3969073

[11] Okunrintemi V Valero-Elizondo J Patrick B Salami J Tibuakuu M Ahmad S Gender differences in patient-reported outcomes among adults with atherosclerotic cardiovascular disease. J Am Heart Assoc. (2018) 7(24):e010498. 10.1161/JAHA.118.010498 30561253 PMC6405598

[12] Solola Nussbaum S Henry S Yong CM Daugherty SL Mehran R Poppas A. Sex-Specific considerations in the presentation, diagnosis, and management of ischemic heart disease: jacc focus seminar 2/7. J Am Coll Cardiol. (2022) 79(14):1398–406. 10.1016/j.jacc.2021.11.065 35393022 PMC9009217

[13] Zuo L Kua Z Wang X Low A Cai X Li S Sex differences in the impact of atherosclerotic cardiovascular risk burden on cognitive health. J Am Heart Assoc. (2025) 14(17):e042741. 10.1161/JAHA.125.042741 40879031 PMC12553448

[14] Appelman Y van Rijn BB Ten Haaf ME Boersma E Peters SA. Sex differences in cardiovascular risk factors and disease prevention. Atherosclerosis. (2015) 241(1):211–8. 10.1016/j.atherosclerosis.2015.01.027 25670232

[15] Hoeper MM Pausch C Grunig E Klose H Staehler G Huscher D Idiopathic pulmonary arterial hypertension phenotypes determined by cluster analysis from the compera registry. J Heart Lung Transplant. (2020) 39(12):1435–44. 10.1016/j.healun.2020.09.011 33082079

[16] Benza RL Miller DP Barst RJ Badesch DB Frost AE McGoon MD. An evaluation of long- term survival from time of diagnosis in pulmonary arterial hypertension from the reveal registry. Chest. (2012) 142(2):448–56. 10.1378/chest.11-1460 22281797

[17] Hurdman J Condliffe R Elliot CA Davies C Hill C Wild JM Aspire registry: assessing the Spectrum of pulmonary hypertension identified at a referral centre. Eur Respir J. (2012) 39(4):945–55. 10.1183/09031936.00078411 21885399

[18] Synn AJ Margerie-Mellon C Jeong SY Rahaghi FN Jhun I Washko GR Vascular remodeling of the small pulmonary arteries and measures of vascular pruning on computed tomography. Pulm Circ. (2021) 11(4):20458940211061284. 10.1177/20458940211061284 34881020 PMC8647266

[19] Dai J Chen H Fang J Wu S Jia Z. Vascular remodeling: the multicellular mechanisms of pulmonary hypertension. Int J Mol Sci. (2025) 26(9):4265. 10.3390/ijms26094265 40362501 PMC12072204

[20] Dieffenbach PB Maracle M Tschumperlin DJ Fredenburgh LE. Mechanobiological feedback in pulmonary vascular disease. Front Physiol. (2018) 9:951. 10.3389/fphys.2018.00951 30090065 PMC6068271

[21] Engström G Lampa E Dekkers K Lin YT Ahlm K Ahlström H Pulmonary function and atherosclerosis in the general population: causal associations and clinical implications. Eur J Epidemiol. (2024) 39(1):35–49. 10.1007/s10654-023-01088-z 38165527 PMC10811042

[22] Stern S. Symptoms other than chest pain may be important in the diagnosis of “silent ischemia,” or “the sounds of silence”. Circulation. (2005) 111(24):e435–7. 10.1161/circulationaha.105.550723 15967853

[23] Fruchart JC Nierman MC Stroes ES Kastelein JJ Duriez P. New risk factors for atherosclerosis and patient risk assessment. Circulation. (2004) 109(23 Suppl 1):Iii15–9. 10.1161/01.CIR.0000131513.33892.5b 15198961

[24] Lau EMT Giannoulatou E Celermajer DS Humbert M. Epidemiology and treatment of pulmonary arterial hypertension. Nat Rev Cardiol. (2017) 14(10):603–14. 10.1038/nrcardio.2017.84 28593996

[25] Lee J Cooke JP. The role of nicotine in the pathogenesis of atherosclerosis. Atherosclerosis. (2011) 215(2):281–3. 10.1016/j.atherosclerosis.2011.01.003 21345436 PMC3755365

[26] Horton WB Love KM Gregory JM Liu Z Barrett EJ. Metabolic and vascular insulin resistance: partners in the pathogenesis of cardiovascular disease in diabetes. Am J Physiol Heart Circ Physiol. (2025) 328(6):H1218–h36. 10.1152/ajpheart.00826.2024 40257392 PMC12172477

[27] Balcan B Akdeniz B Peker Y The Turcosact C. Obstructive sleep apnea and pulmonary hypertension: a chicken-and-egg relationship. J Clin Med. (2024) 13(10):2961. 10.3390/jcm13102961 38792502 PMC11122166

[28] Ejikeme C Safdar Z. Exploring the pathogenesis of pulmonary vascular disease. Front Med (Lausanne). (2024) 11:1402639. 10.3389/fmed.2024.1402639 39050536 PMC11267418

[29] Phillips CL O'Driscoll DM. Hypertension and obstructive sleep apnea. Nat Sci Sleep. (2013) 5:43–52. 10.2147/nss.S34841 23750107 PMC3666153

[30] Seegers LM Araki M Nakajima A Yonetsu T Minami Y Ako J Sex differences in culprit plaque characteristics among different age groups in patients with acute coronary syndromes. Circ Cardiovasc Interv. (2022) 15(6):e011612. 10.1161/circinterventions.121.011612 35652353

[31] Laban D Kattan A Ait-Abdellah L Krishna H Le Master E Levitan I. Sex differences in features of atherosclerotic plaques as revealed by Various imaging techniques: historical review. Front Physiol. (2025) 16:1579885. 10.3389/fphys.2025.1579885 40491450 PMC12146876

[32] Stanhewicz AE Wenner MM Stachenfeld NS. Sex differences in endothelial function important to vascular health and overall cardiovascular disease risk across the lifespan. Am J Physiol Heart Circ Physiol. (2018) 315(6):H1569–H88. 10.1152/ajpheart.00396.2018 30216121 PMC6734083

[33] Ma JI Owunna N Mounsey LA Jiang NM Huo X Zern E Sex differences in pulmonary hypertension and associated right ventricular dysfunction. Am J Cardiol. (2026) 259:173–8. 10.1016/j.amjcard.2025.09.046 41043601 PMC12970616

[34] Manrique-Acevedo C Chinnakotla B Padilla J Martinez-Lemus LA Gozal D. Obesity and cardiovascular disease in women. Int J Obes (Lond). (2020) 44(6):1210–26. 10.1038/s41366-020-0548-0 32066824 PMC7478041

[35] Garcia M Mulvagh SL Merz CN Buring JE Manson JE. Cardiovascular disease in women: clinical perspectives. Circ Res. (2016) 118(8):1273–93. 10.1161/circresaha.116.307547 27081110 PMC4834856

[36] Lakshmanan S Jankowich M Wu WC Blackshear C Abbasi S Choudhary G. Gender differences in risk factors associated with pulmonary artery systolic pressure, heart failure, and mortality in blacks: jackson heart study. J Am Heart Assoc. (2020) 9(1):e013034. 10.1161/jaha.119.013034 31902323 PMC6988159

[37] Varghese M Griffin C McKernan K Eter L Lanzetta N Agarwal D Sex differences in inflammatory responses to adipose tissue lipolysis in diet-induced obesity. Endocrinology. (2019) 160(2):293–312. 10.1210/en.2018-00797 30544158 PMC6330175

[38] Poret JM Gaudet DA Braymer HD Primeaux SD. Sex differences in markers of metabolic syndrome and adipose tissue inflammation in obesity-prone, osborne-Mendel and obesity-resistant, S5b/pl rats. Life Sci. (2021) 273:119290. 10.1016/j.lfs.2021.119290 33662430 PMC9594853

[39] Halstead KM Wetzel EM Cho JL Stanhewicz AE. Sex differences in oxidative stress- mediated reductions in microvascular endothelial function in young adult E-cigarette users. Hypertension. (2023) 80(12):2641–9. 10.1161/HYPERTENSIONAHA.123.21684 37800370 PMC10848654

[40] Ventetuolo CE Sherman-Roe AE. Sex differences in pulmonary (arterial) hypertension: does it matter? Curr Opin Pulm Med. (2025) 31(5):411–28. 10.1097/mcp.0000000000001197 40767089 PMC12331139

[41] Fairweather D Frisancho-Kiss S Rose NR. Sex differences in autoimmune disease from a pathological perspective. Am J Pathol. (2008) 173(3):600–9. 10.2353/ajpath.2008.071008 18688037 PMC2527069

[42] Huertas A Perros F Tu L Cohen-Kaminsky S Montani D Dorfmuller P Immune dysregulation and endothelial dysfunction in pulmonary arterial hypertension: a Complex interplay. Circulation. (2014) 129(12):1332–40. 10.1161/CIRCULATIONAHA.113.004555 24664216

[43] Sheifer SE Canos MR Weinfurt KP Arora UK Mendelsohn FO Gersh BJ Sex differences in coronary artery size assessed by intravascular ultrasound. Am Heart J. (2000) 139(4):649–53. 10.1016/s0002-8703(00)90043-7 10740147

[44] Wentzel JJ Papafaklis MI Antoniadis AP Takahashi S Cefalo NV Cormier M Sex- related differences in plaque characteristics and endothelial shear stress related plaque- progression in human coronary arteries. Atherosclerosis. (2022) 342:9–18. 10.1016/j.atherosclerosis.2021.12.013 34999306

[45] Wentzel JJ Bos D White SJ van der Heiden K Kavousi M Evans PC. Sex-Related differences in coronary and carotid vessel geometry, plaque composition and shear stress obtained from imaging. Atherosclerosis. (2024) 395:117616. 10.1016/j.atherosclerosis.2024.117616 38944895

[46] Shelburne NJ Nian H Beck GJ Casanova NG Desai AA DuBrock HM Association of male sex with worse right ventricular function and survival in pulmonary hypertension in the redefining pulmonary hypertension through pulmonary vascular disease phenomics cohort. CHEST Pulm. (2024) 2(3):100046. 10.1016/j.chpulm.2024.100046 39524046 PMC11548889

[47] Tello K Richter MJ Yogeswaran A Ghofrani HA Naeije R Vanderpool R Sex differences in right ventricular-pulmonary arterial coupling in pulmonary arterial hypertension. Am J Respir Crit Care Med. (2020) 202(7):1042–6. 10.1164/rccm.202003-0807LE 32501730 PMC7528786

[48] Liu A Philip J Vinnakota KC Van den Bergh F Tabima DM Hacker T Estrogen maintains mitochondrial content and function in the right ventricle of rats with pulmonary hypertension. Physiol Rep. (2017) 5(6):e13157. 10.14814/phy2.13157 28320896 PMC5371553

[49] Kjellstrom B Nisell M Kylhammar D Bartfay SE Ivarsson B Radegran G Sex- specific differences and survival in patients with idiopathic pulmonary arterial hypertension 2008–2016. ERJ Open Res. (2019) 5(3):00075–2019. 10.1183/23120541.00075-2019 31423450 PMC6689671

[50] Guetta V Cannon RO. 3rd. Cardiovascular effects of estrogen and lipid-lowering therapies in postmenopausal women. Circulation. (1996) 93(10):1928–37. 10.1161/01.cir.93.10.1928 8635273

[51] Gungor F Kalelioglu I Turfanda A. Vascular effects of estrogen and progestins and risk of coronary artery disease: importance of timing of estrogen treatment. Angiology. (2009) 60(3):308–17. 10.1177/0003319708318377 18505742

[52] SenthilKumar G Katunaric B Bordas-Murphy H Sarvaideo J Freed JK. Estrogen and the vascular endothelium: the unanswered questions. Endocrinology. (2023) 164(6):bqad079. 10.1210/endocr/bqad079 37207450 PMC10230790

[53] DesJardin JT Kime N Kolaitis NA Kronmal RA Lammi MR Mathai SC Investigating the “sex paradox” in pulmonary arterial hypertension: results from the pulmonary hypertension association registry (phar). J Heart Lung Transplant. (2024) 43(6):901–10. 10.1016/j.healun.2024.02.004 38360160 PMC11500812

[54] Umar S Rabinovitch M Eghbali M. Estrogen paradox in pulmonary hypertension: current controversies and future perspectives. Am J Respir Crit Care Med. (2012) 186(2):125–31. 10.1164/rccm.201201-0058PP 22561960 PMC3406082

[55] Hester J Ventetuolo C Lahm T. Sex, gender, and sex hormones in pulmonary hypertension and right ventricular failure. Compr Physiol. (2019) 10(1):125–70. 10.1002/cphy.c190011 31853950 PMC7338988

[56] Umar S Iorga A Matori H Nadadur RD Li J Maltese F Estrogen rescues preexisting severe pulmonary hypertension in rats. Am J Respir Crit Care Med. (2011) 184(6):715–23. 10.1164/rccm.201101-0078OC 21700911 PMC3208600

[57] Billon-Galés A Fontaine C Douin-Echinard V Delpy L Berges H Calippe B Endothelial estrogen receptor-alpha plays a crucial role in the atheroprotective action of 17beta-estradiol in low-density lipoprotein receptor-deficient mice. Circulation. (2009) 120(25):2567–76. 10.1161/circulationaha.109.898445 19996016

[58] Chen Z Yuhanna IS Galcheva-Gargova Z Karas RH Mendelsohn ME Shaul PW. Estrogen receptor alpha mediates the nongenomic activation of endothelial nitric oxide synthase by estrogen. J Clin Invest. (1999) 103(3):401–6. 10.1172/jci5347 9927501 PMC407904

[59] MacRitchie AN Jun SS Chen Z German Z Yuhanna IS Sherman TS Estrogen upregulates endothelial nitric oxide synthase gene expression in fetal pulmonary artery endothelium. Circ Res. (1997) 81(3):355–62. 10.1161/01.res.81.3.355 9285637

[60] Sun Y Sangam S Guo Q Wang J Tang H Black SM Sex differences, estrogen metabolism and signaling in the development of pulmonary arterial hypertension. Front Cardiovasc Med. (2021) 8:719058. 10.3389/fcvm.2021.719058 34568460 PMC8460911

[61] Chen Y Li Y Leng B Cao C Wu G Ye S Lncrna myoslid contributes to ph via targeting Bmpr2 signaling in pulmonary artery smooth muscle cell. Vascul Pharmacol. (2024) 157:107439. 10.1016/j.vph.2024.107439 39549862

[62] Evans JD Girerd B Montani D Wang XJ Galiè N Austin ED Bmpr2 mutations and survival in pulmonary arterial hypertension: an individual participant data meta-analysis. Lancet Respir Med. (2016) 4(2):129–37. 10.1016/s2213-2600(15)00544-5 26795434 PMC4737700

[63] White K Johansen AK Nilsen M Ciuclan L Wallace E Paton L Activity of the estrogen-metabolizing enzyme cytochrome P450 1b1 influences the development of pulmonary arterial hypertension. Circulation. (2012) 126(9):1087–98. 10.1161/CIRCULATIONAHA.111.062927 22859684

[64] Hood KY Montezano AC Harvey AP Nilsen M MacLean MR Touyz RM. Nicotinamide adenine dinucleotide phosphate oxidase-mediated redox signaling and vascular remodeling by 16alpha-hydroxyestrone in human pulmonary artery cells: implications in pulmonary arterial hypertension. Hypertension. (2016) 68(3):796–808. 10.1161/HYPERTENSIONAHA.116.07668 27402919 PMC4978604

[65] Alencar AKN Montes GC Costa DG Mendes LVP Silva AMS Martinez ST Cardioprotection induced by activation of gper in ovariectomized rats with pulmonary hypertension. J Gerontol A Biol Sci Med Sci. (2018) 73(9):1158–66. 10.1093/gerona/gly068 29790948 PMC6093348

[66] Lahm T Crisostomo PR Markel TA Wang M Wang Y Tan J Selective estrogen receptor-alpha and estrogen receptor-Beta agonists rapidly decrease pulmonary artery vasoconstriction by a nitric oxide-dependent mechanism. Am J Physiol Regul Integr Comp Physiol. (2008) 295(5):R1486–93. 10.1152/ajpregu.90667.2008 18832085 PMC2584849

[67] Fredette NC Meyer MR Prossnitz ER. Role of gper in estrogen-dependent nitric oxide formation and vasodilation. J Steroid Biochem Mol Biol. (2018) 176:65–72. 10.1016/j.jsbmb.2017.05.006 28529128 PMC5694388

[68] Manson JE Allison MA Rossouw JE Carr JJ Langer RD Hsia J Estrogen therapy and coronary-artery calcification. N Engl J Med. (2007) 356(25):2591–602. 10.1056/NEJMoa071513 17582069

[69] Torres-Estay V Carreno DV San Francisco IF Sotomayor P Godoy AS Smith GJ. Androgen receptor in human endothelial cells. J Endocrinol. (2015) 224(3):R131–7. 10.1530/JOE-14-0611 25563353 PMC4700832

[70] Kelly DM Jones TH. Testosterone: a vascular hormone in health and disease. J Endocrinol. (2013) 217(3):R47–71. 10.1530/joe-12-0582 23549841

[71] Jones RD Pugh PJ Jones TH Channer KS. The vasodilatory action of testosterone: a potassium-channel opening or a calcium antagonistic action? Br J Pharmacol. (2003) 138(5):733–44. 10.1038/sj.bjp.0705141 12642373 PMC1573742

[72] Zgliczynski S Ossowski M Slowinska-Srzednicka J Brzezinska A Zgliczynski W Soszynski P Effect of testosterone replacement therapy on lipids and lipoproteins in hypogonadal and elderly men. Atherosclerosis. (1996) 121(1):35–43. 10.1016/0021-9150(95)05673-4 8678922

[73] Di Lodovico E Facondo P Delbarba A Pezzaioli LC Maffezzoni F Cappelli C Testosterone, hypogonadism, and heart failure. Circ Heart Fail. (2022) 15(7):e008755. 10.1161/circheartfailure.121.008755 35392658

[74] Davis SR. Testosterone and the heart: friend or foe? Climacteric. (2024) 27(1):53–9. 10.1080/13697137.2023.2250252 37666273

[75] Hemnes AR Maynard KB Champion HC Gleaves L Penner N West J Testosterone negatively regulates right ventricular load stress responses in mice. Pulm Circ. (2012) 2(3):352–8. 10.4103/2045-8932.101647 23130103 PMC3487303

[76] Rowell KO Hall J Pugh PJ Jones TH Channer KS Jones RD. Testosterone acts as an efficacious vasodilator in isolated human pulmonary arteries and veins: evidence for a biphasic effect at physiological and supra-physiological concentrations. J Endocrinol Invest. (2009) 32(9):718–23. 10.1007/BF03346526 19535892

[77] Liang Z Zhu J Chen L Xu Y Yang Y Hu R Is androgen deprivation therapy for prostate cancer associated with cardiovascular disease? A meta-analysis and systematic review. Andrology. (2020) 8(3):559–74. 10.1111/andr.12731 31743594

[78] Khorram AA Pourasgharian R Shams AS Toufani S Mostafaei M Khademi R Androgen deprivation therapy use and the risk of heart failure in patients with prostate cancer: a systematic review and meta-analysis. BMC Cardiovasc Disord. (2024) 24(1):756. 10.1186/s12872-024-04421-w 39736562 PMC11684147

[79] Jaiswal V Sawhney A Nebuwa C Borra V Deb N Halder A Association between testosterone replacement therapy and cardiovascular outcomes: a meta-analysis of 30 randomized controlled trials. Prog Cardiovasc Dis. (2024) 85:45–53. 10.1016/j.pcad.2024.04.001 38589271

[80] Harada N Sasano H Murakami H Ohkuma T Nagura H Takagi Y. Localized expression of aromatase in human vascular tissues. Circ Res. (1999) 84(11):1285–91. 10.1161/01.res.84.11.1285 10364566

[81] Lahm T Kawut SM. Inhibiting oestrogen signalling in pulmonary arterial hypertension: sex, drugs and research. Eur Respir J. (2017) 50(2):1700983. 10.1183/13993003.00983-2017 28775051

[82] Natarajan P Pampana A Graham SE Ruotsalainen SE Perry JA de Vries PS Chromosome Xq23 is associated with lower atherogenic lipid concentrations and favorable cardiometabolic indices. Nat Commun. (2021) 12(1):2182. 10.1038/s41467-021-22339-1 33846329 PMC8042019

[83] Zore T Palafox M Reue K. Sex differences in obesity, lipid metabolism, and inflammation-a role for the sex chromosomes? Mol Metab. (2018) 15:35–44. 10.1016/j.molmet.2018.04.003 29706320 PMC6066740

[84] Bianchi I Lleo A Gershwin ME Invernizzi P. The X chromosome and immune associated genes. J Autoimmun. (2012) 38(2-3):J187–92. 10.1016/j.jaut.2011.11.012 22178198

[85] Spiering AE Groenheide PJ Mokry M Onland-Moret NC Civelek M Reue K Sex chromosomes and cardiovascular disease. Eur J Prev Cardiol. (2025). 10.1093/eurjpc/zwaf224 40231569

[86] Youness A Miquel CH Guéry JC. Escape from X chromosome inactivation and the female predominance in autoimmune diseases. Int J Mol Sci. (2021) 22(3):1114. 10.3390/ijms22031114 33498655 PMC7865432

[87] Chlamydas S Markouli M Strepkos D Piperi C. Epigenetic mechanisms regulate sex- specific bias in disease manifestations. J Mol Med (Berl). (2022) 100(8):1111–23. 10.1007/s00109-022-02227-x 35764820 PMC9244100

[88] Sakkas LI Chikanza IC. Sex bias in immune response: it is time to include the sex Variable in studies of autoimmune rheumatic diseases. Rheumatol Int. (2024) 44(2):203–9. 10.1007/s00296-023-05446-8 37716925

[89] Munjas J Vladimirov S Ratkovic T Comi L Giglione C Tanaskovic I Toward precision medicine in atherosclerotic cardiovascular disease: insights from omics data into sex differences. Curr Atheroscler Rep. (2025) 28(1):5. 10.1007/s11883-025-01380-1 41460411 PMC12748300

[90] Li X Zhang Q Shi Q Liu Y Zhao K Shen Q Demethylase Kdm6a epigenetically promotes il-6 and ifn-Beta production in macrophages. J Autoimmun. (2017) 80:85–94. 10.1016/j.jaut.2017.02.007 28284523

[91] Kesavardhana S Samir P Zheng M Malireddi RKS Karki R Sharma BR Ddx3x coordinates host defense against influenza virus by activating the Nlrp3 inflammasome and type I interferon response. J Biol Chem. (2021) 296:100579. 10.1016/j.jbc.2021.100579 33766561 PMC8081917

[92] Girerd B Weatherald J Montani D Humbert M. Heritable pulmonary hypertension: from bench to bedside. Eur Respir Rev. (2017) 26(145):170037. 10.1183/16000617.0037-2017 28877973 PMC9489013

[93] Austin ED Loyd JE. The genetics of pulmonary arterial hypertension. Circ Res. (2014) 115(1):189–202. 10.1161/CIRCRESAHA.115.303404 24951767 PMC4137413

[94] Yan L Cogan JD Hedges LK Nunley B Hamid R Austin ED. The Y chromosome regulates Bmpr2 expression via sry: a possible reason “why” fewer males develop pulmonary arterial hypertension. Am J Respir Crit Care Med. (2018) 198(12):1581–3. 10.1164/rccm.201802-0308LE 30252494 PMC6298637

[95] Austin ED Cogan JD West JD Hedges LK Hamid R Dawson EP Alterations in oestrogen metabolism: implications for higher penetrance of familial pulmonary arterial hypertension in females. Eur Respir J. (2009) 34(5):1093–9. 10.1183/09031936.00010409 19357154 PMC3742124

[96] Chen X Talati M Fessel JP Hemnes AR Gladson S French J Estrogen metabolite 16alpha-hydroxyestrone exacerbates bone morphogenetic protein receptor type ii-associated pulmonary arterial hypertension through microrna-29-mediated modulation of cellular metabolism. Circulation. (2016) 133(1):82–97. 10.1161/CIRCULATIONAHA.115.016133 26487756 PMC4698046

[97] Jaliawala HA Parmar M Summers K Bernardo RJ. A second hit? Pulmonary arterial hypertension, Bmpr2 mutation, and exposure to prescription amphetamines. Pulm Circ. (2022) 12(1):e12053. 10.1002/pul2.12053 35506068 PMC9052970

[98] Hamid R Cogan JD Hedges LK Austin E Phillips JA 3rd Newman JH Penetrance of pulmonary arterial hypertension is modulated by the expression of normal Bmpr2 allele. Hum Mutat. (2009) 30(4):649–54. 10.1002/humu.20922 19206171 PMC2663001

[99] Krzyżewska A Kurakula K. Sex dimorphism in pulmonary arterial hypertension associated with autoimmune diseases. Arterioscler Thromb Vasc Biol. (2024) 44(10):2169–90. 10.1161/atvbaha.124.320886 39145392

[100] Yeh FC Chen CN Xie CY Baxan N Zhao L Ashek A Tlr7/8 activation induces autoimmune vasculopathy and causes severe pulmonary arterial hypertension. Eur Respir J. (2023) 62(1):2300204. 10.1183/13993003.00204-2023 37290790 PMC10356963

[101] Itoh Y Mackie R Kampf K Domadia S Brown JD O'Neill R Four core genotypes mouse model: localization of the sry transgene and bioassay for testicular hormone levels. BMC Res Notes. (2015) 8:69. 10.1186/s13104-015-0986-2 25870930 PMC4354741

[102] Li J Chen X McClusky R Ruiz-Sundstrom M Itoh Y Umar S The number of X chromosomes influences protection from cardiac ischaemia/reperfusion injury in mice: one X is better than two. Cardiovasc Res. (2014) 102(3):375–84. 10.1093/cvr/cvu064 24654234 PMC4030514

[103] Arnold AP. Four core genotypes and Xy* mouse models: update on impact on sabv research. Neurosci Biobehav Rev. (2020) 119:1–8. 10.1016/j.neubiorev.2020.09.021 32980399 PMC7736196

[104] van Rooij E Sutherland LB Thatcher JE DiMaio JM Naseem RH Marshall WS Dysregulation of micrornas after myocardial infarction reveals a role of mir-29 in cardiac fibrosis. Proc Natl Acad Sci U S A. (2008) 105(35):13027–32. 10.1073/pnas.0805038105 18723672 PMC2529064

[105] Courboulin A Paulin R Giguere NJ Saksouk N Perreault T Meloche J Role for mir- 204 in human pulmonary arterial hypertension. J Exp Med. (2011) 208(3):535–48. 10.1084/jem.20101812 21321078 PMC3058572

[106] Pullamsetti SS Kojonazarov B Storn S Gall H Salazar Y Wolf J Lung cancer- associated pulmonary hypertension: role of microenvironmental inflammation based on tumor cell-immune cell cross-talk. Sci Transl Med. (2017) 9(416):eaai9048. 10.1126/scitranslmed.aai9048 29141888

[107] Arnold AP Cassis LA Eghbali M Reue K Sandberg K. Sex hormones and sex chromosomes cause sex differences in the development of cardiovascular diseases. Arterioscler Thromb Vasc Biol. (2017) 37(5):746–56. 10.1161/atvbaha.116.307301 28279969 PMC5437981

[108] Hemnes AR Brittain EL Trammell AW Fessel JP Austin ED Penner N Evidence for right ventricular lipotoxicity in heritable pulmonary arterial hypertension. Am J Respir Crit Care Med. (2014) 189(3):325–34. 10.1164/rccm.201306-1086OC 24274756 PMC3977729

[109] Wang Z Klipfell E Bennett BJ Koeth R Levison BS Dugar B Gut Flora metabolism of phosphatidylcholine promotes cardiovascular disease. Nature. (2011) 472(7341):57–63. 10.1038/nature09922 21475195 PMC3086762

[110] Koeth RA Wang Z Levison BS Buffa JA Org E Sheehy BT Intestinal Microbiota metabolism of L-carnitine, a nutrient in red meat, promotes atherosclerosis. Nat Med. (2013) 19(5):576–85. 10.1038/nm.3145 23563705 PMC3650111

[111] Kim S Rigatto K Gazzana MB Knorst MM Richards EM Pepine CJ Altered gut microbiome profile in patients with pulmonary arterial hypertension. Hypertension. (2020) 75(4):1063–71. 10.1161/HYPERTENSIONAHA.119.14294 32088998 PMC7067661

[112] Mauvais-Jarvis F Bairey Merz N Barnes PJ Brinton RD Carrero JJ DeMeo DL Sex and gender: modifiers of health, disease, and medicine. Lancet. (2020) 396(10250):565–82. 10.1016/S0140-6736(20)31561-0 32828189 PMC7440877

[113] Napoli C Benincasa G Loscalzo J. Epigenetic inheritance underlying pulmonary arterial hypertension. Arterioscler Thromb Vasc Biol. (2019) 39(4):653–64. 10.1161/ATVBAHA.118.312262 30727752 PMC6436974

[114] Frump AL Goss KN Vayl A Albrecht M Fisher A Tursunova R Estradiol improves right ventricular function in rats with severe angioproliferative pulmonary hypertension: effects of endogenous and exogenous sex hormones. Am J Physiol Lung Cell Mol Physiol. (2015) 308(9):L873–90. 10.1152/ajplung.00006.2015 25713318 PMC4421786

[115] Lahm T Frump AL Albrecht ME Fisher AJ Cook TG Jones TJ 17beta-Estradiol Mediates superior adaptation of right ventricular function to acute strenuous exercise in female rats with severe pulmonary hypertension. Am J Physiol Lung Cell Mol Physiol. (2016) 311(2):L375–88. 10.1152/ajplung.00132.2016 27288487 PMC5142461

[116] Plump AS Smith JD Hayek T Aalto-Setälä K Walsh A Verstuyft JG Severe hypercholesterolemia and atherosclerosis in apolipoprotein E-deficient mice created by homologous recombination in es cells. Cell. (1992) 71(2):343–53. 10.1016/0092-8674(92)90362-g 1423598

[117] Ishibashi S Brown MS Goldstein JL Gerard RD Hammer RE Herz J. Hypercholesterolemia in low density lipoprotein receptor knockout mice and its reversal by adenovirus-mediated gene delivery. J Clin Invest. (1993) 92(2):883–93. 10.1172/jci116663 8349823 PMC294927

[118] Voelkel NF Tuder RM. Hypoxia-Induced pulmonary vascular remodeling: a model for what human disease? J Clin Invest. (2000) 106(6):733–8. 10.1172/jci11144 10995781 PMC381402

[119] Stenmark KR Meyrick B Galie N Mooi WJ McMurtry IF. Animal models of pulmonary arterial hypertension: the hope for etiological discovery and pharmacological cure. Am J Physiol Lung Cell Mol Physiol. (2009) 297(6):L1013–32. 10.1152/ajplung.00217.2009 19748998

[120] Chaudhary KR Deng Y Yang A Cober ND Stewart DJ. Penetrance of severe pulmonary arterial hypertension in response to vascular endothelial growth factor receptor 2 blockade in a genetically prone rat model is reduced by female sex. J Am Heart Assoc. (2021) 10(15):e019488. 10.1161/jaha.120.019488 34315227 PMC8475703

[121] Boucherat O Agrawal V Lawrie A Bonnet S. The latest in animal models of pulmonary hypertension and right ventricular failure. Circ Res. (2022) 130(9):1466–86. 10.1161/CIRCRESAHA.121.319971 35482834 PMC9060385

[122] Gistera A Ketelhuth DFJ Malin SG Hansson GK. Animal models of atherosclerosis- supportive notes and tricks of the trade. Circ Res. (2022) 130(12):1869–87. 10.1161/CIRCRESAHA.122.320263 35679358

[123] Caligiuri G Nicoletti A Zhou X Törnberg I Hansson GK. Effects of sex and age on atherosclerosis and autoimmunity in apoe-deficient mice. Atherosclerosis. (1999) 145(2):301–8. 10.1016/s0021-9150(99)00081-7 10488957

[124] Bourassa PA Milos PM Gaynor BJ Breslow JL Aiello RJ. Estrogen reduces atherosclerotic lesion development in apolipoprotein E-deficient mice. Proc Natl Acad Sci U S A. (1996) 93(19):10022–7. 10.1073/pnas.93.19.10022 8816744 PMC38329

[125] Christ C Ocskay Z Kovács G Jakus Z. Characterization of atherosclerotic mice reveals a sex-dependent susceptibility to plaque calcification but No Major changes in the lymphatics in the arterial wall. Int J Mol Sci. (2024) 25(7):4046. 10.3390/ijms25074046 38612867 PMC11012298

[126] Umar S Partow-Navid R Ruffenach G Iorga A Moazeni S Eghbali M. Severe pulmonary hypertension in aging female apolipoprotein E-deficient mice is rescued by estrogen replacement therapy. Biol Sex Differ. (2017) 8:9. 10.1186/s13293-017-0129-7 28344760 PMC5360087

[127] Bal E Ilgin S Atli O Ergun B Sirmagul B. The effects of gender difference on monocrotaline-induced pulmonary hypertension in rats. Hum Exp Toxicol. (2013) 32(7):766–74. 10.1177/0960327113477874 23821593

[128] Kwan ED Hardie BA Garcia KM Mu H Wang TM Valdez-Jasso D. Sex-Dependent remodeling of right ventricular function in a rat model of pulmonary arterial hypertension. Am J Physiol Heart Circ Physiol. (2024) 327(2):H351–h63. 10.1152/ajpheart.00098.2024 38847755 PMC11932540

[129] Neupane B Sydykov A Pradhan K Vroom C Herden C Karnati S Influence of gender in monocrotaline and chronic hypoxia induced pulmonary hypertension in obese rats and mice. Respir Res. (2020) 21(1):136. 10.1186/s12931-020-01394-0 32493503 PMC7268383

[130] Hautefort A Mendes-Ferreira P Sabourin J Manaud G Bertero T Rucker-Martin C Bmpr2 mutant rats develop pulmonary and cardiac characteristics of pulmonary arterial hypertension. Circulation. (2019) 139(7):932–48. 10.1161/CIRCULATIONAHA.118.033744 30586714

[131] Erewele EO Castellon M Loya O Marshboom G Schwartz A Yerlioglu K Hypoxia-induced pulmonary hypertension upregulates enos and tgf-beta contributing to sex-linked differences in *BMPR2*^+/R899X^ mutant mice. Pulm Circ. (2022) 12(4):e12163. 10.1002/pul2.12163 36484056 PMC9722973

[132] Dignam JP Sharma S Stasinopoulos I MacLean MR. Pulmonary arterial hypertension: sex matters. Br J Pharmacol. (2024) 181(7):938–66. 10.1111/bph.16277 37939796

[133] North BJ Sinclair DA. The intersection between aging and cardiovascular disease. Circ Res. (2012) 110(8):1097–108. 10.1161/CIRCRESAHA.111.246876 22499900 PMC3366686

[134] Donato AJ Machin DR Lesniewski LA. Mechanisms of dysfunction in the aging vasculature and role in age-related disease. Circ Res. (2018) 123(7):825–48. 10.1161/CIRCRESAHA.118.312563 30355078 PMC6207260

[135] Ungvari Z Tarantini S Donato AJ Galvan V Csiszar A. Mechanisms of vascular aging. Circ Res. (2018) 123(7):849–67. 10.1161/CIRCRESAHA.118.311378 30355080 PMC6248882

[136] Oh JE Jung C Yoon YS. Human induced pluripotent stem cell-derived vascular cells: recent progress and future directions. J Cardiovasc Dev Dis. (2021) 8(11):148. 10.3390/jcdd8110148 34821701 PMC8622843

[137] Budeus B Kroepel C Stasch LM Klein D. Matrix-Free human lung organoids derived from induced pluripotent stem cells to model lung injury. Stem Cell Res Ther. (2024) 15(1):468. 10.1186/s13287-024-04106-3 39696649 PMC11657174

[138] Kurokawa YK Yin RT Shang MR Shirure VS Moya ML George SC. Human induced pluripotent stem cell-derived endothelial cells for three-dimensional microphysiological systems. Tissue Eng Part C Methods. (2017) 23(8):474–84. 10.1089/ten.TEC.2017.0133 28622076 PMC5567879

[139] Jin KT Du WL Lan HR Liu YY Mao CS Du JL Development of humanized mouse with patient-derived Xenografts for cancer immunotherapy studies: a comprehensive review. Cancer Sci. (2021) 112(7):2592–606. 10.1111/cas.14934 33938090 PMC8253285

[140] Schermuly RT Ghofrani HA Wilkins MR Grimminger F. Mechanisms of disease: pulmonary arterial hypertension. Nat Rev Cardiol. (2011) 8(8):443–55. 10.1038/nrcardio.2011.87 21691314 PMC7097518

[141] Gimbrone MA J García-Cardeña G. Endothelial cell dysfunction and the pathobiology of atherosclerosis. Circ Res. (2016) 118(4):620–36. 10.1161/circresaha.115.306301 26892962 PMC4762052

[142] Prapa M McCarthy KP Dimopoulos K Sheppard MN Krexi D Swan L Histopathology of the great vessels in patients with pulmonary arterial hypertension in association with congenital heart disease: large pulmonary arteries matter too. Int J Cardiol. (2013) 168(3):2248–54. 10.1016/j.ijcard.2013.01.210 23453874

[143] Schamroth Pravda N Karny-Rahkovich O Shiyovich A Schamroth Pravda M Rapeport N Vaknin-Assa H Coronary artery disease in women: a comprehensive appraisal. J Clin Med. (2021) 10(20):4664. 10.3390/jcm10204664 34682787 PMC8541551

[144] Alturaif N Attanasio U Mercurio V. Pulmonary arterial hypertension: sex-specific differences and outcomes. Ther Adv Respir Dis. (2025) 19:17534666251350493. 10.1177/17534666251350493 40539543 PMC12181702

[145] Redfield MM Rodeheffer RJ Jacobsen SJ Mahoney DW Bailey KR Burnett JC Jr. Plasma brain natriuretic peptide concentration: impact of age and gender. J Am Coll Cardiol. (2002) 40(5):976–82. 10.1016/s0735-1097(02)02059-4 12225726

[146] Lam CS Cheng S Choong K Larson MG Murabito JM Newton-Cheh C Influence of sex and hormone Status on circulating natriuretic peptides. J Am Coll Cardiol. (2011) 58(6):618–26. 10.1016/j.jacc.2011.03.042 21798425 PMC3170816

[147] Myhre PL Claggett B Yu B Skali H Solomon SD Rosjo H Sex and race differences in N-terminal pro-B-type natriuretic peptide concentration and absolute risk of heart failure in the community. JAMA Cardiol. (2022) 7(6):623–31. 10.1001/jamacardio.2022.0680 35476049 PMC9047747

[148] Cao M Pierce AE Norman MS Thakur B Diercks K Hale C Systematic review of sex-specific high sensitivity cardiac troponin I and T thresholds. Clin Ther. (2024) 46(12):988–94. 10.1016/j.clinthera.2024.09.025 39505672

[149] Tossavainen E Soderberg S Gronlund C Gonzalez M Henein MY Lindqvist P. Pulmonary artery acceleration time in identifying pulmonary hypertension patients with raised pulmonary vascular resistance. Eur Heart J Cardiovasc Imaging. (2013) 14(9):890–7. 10.1093/ehjci/jes309 23295626

[150] Farmakis IT Demerouti E Karyofyllis P Karatasakis G Stratinaki M Tsiapras D Echocardiography in pulmonary arterial hypertension: is it time to reconsider its prognostic utility? J Clin Med. (2021) 10(13):2826. 10.3390/jcm10132826 34206876 PMC8268493

[151] Eckstein J Korperich H Weber OM Burchert W Pugachov V Demydiuk O Assessment of cmr feature-tracking age- and sex-dependent right ventricular strain in a healthy Caucasian cohort. J Cardiovasc Transl Res. (2025) 18(1):146–57. 10.1007/s12265-024-10557-z 39292408 PMC11885332

[152] Synn AJ Li W San Jose Estepar R Washko GR O'Connor GT Tsao CW Pulmonary vascular pruning on computed tomography and risk of death in the framingham heart study. Am J Respir Crit Care Med. (2021) 203(2):251–4. 10.1164/rccm.202005-1671LE 32926788 PMC7874407

[153] Watts JR J Sonavane SK Snell-Bergeon J Nath H. Visual scoring of coronary artery calcification in lung cancer screening computed tomography: association with all-cause and cardiovascular mortality risk. Coron Artery Dis. (2015) 26(2):157–62. 10.1097/MCA.0000000000000189 25370000

[154] Humbert M Kovacs G Hoeper MM Badagliacca R Berger RMF Brida M 2022 Esc/ers guidelines for the diagnosis and treatment of pulmonary hypertension. Eur Heart J. (2022) 43(38):3618–731. 10.1093/eurheartj/ehac237 36017548

[155] Mbau L Gulati M Ngunga M Shah J Gitura B Barasa F Sex differences in clinical characteristics, treatment, and outcomes of cardiovascular disease: Kenya heart registry analysis. JACC Adv. (2025) 5(1):102466. 10.1016/j.jacadv.2025.102466 41418695 PMC12775952

[156] Kiss PAJ Uijl A de Boer AR Duk TCX Grobbee DE Hollander M Sex differences in the intensity of statin prescriptions at initiation in a primary care setting. Heart. (2024) 110(15):981–7. 10.1136/heartjnl-2023-323722 38580433

[157] Vartak N Ong C Huang X Weintraub S Parikh N Rosen S Gender differences in lipid management, ldl-C goal attainment, and prescribing practices. J Clin Lipidol. (2025). 10.1016/j.jacl.2025.12.008 41494938

[158] Brown CJ Chang LS Hosomura N Malmasi S Morrison F Shubina M Assessment of sex disparities in nonacceptance of statin therapy and low-density lipoprotein cholesterol levels among patients at high cardiovascular risk. JAMA Netw Open. (2023) 6(2):e231047. 10.1001/jamanetworkopen.2023.1047 36853604 PMC9975905

[159] Galema-Boers AMH Mulder J Steward K van Lennep JE R. Sex differences in efficacy and safety of Pcsk9 monoclonal antibodies: a real-world registry. Atherosclerosis. (2023) 384:117108. 10.1016/j.atherosclerosis.2023.03.013 37059655

[160] Paquette M Faubert S Saint-Pierre N Baass A Bernard S. Sex differences in ldl-C response to Pcsk9 inhibitors: a real world experience. J Clin Lipidol. (2023) 17(1):142–9. 10.1016/j.jacl.2022.12.002 36641299

[161] Rivera FB Cha SW Aparece JP Rocimo A Ong BA Golbin JM Sex differences in cardiovascular outcomes and cholesterol-lowering efficacy of Pcsk9 inhibitors: systematic review and meta-analysis. JACC Adv. (2023) 2(9):100669. 10.1016/j.jacadv.2023.100669 38938736 PMC11198239

[162] Gabler NB French B Strom BL Liu Z Palevsky HI Taichman DB Race and sex differences in response to endothelin receptor antagonists for pulmonary arterial hypertension. Chest. (2012) 141(1):20–6. 10.1378/chest.11-0404 21940766 PMC5991545

[163] Kozu K Sugimura K Aoki T Tatebe S Yamamoto S Yaoita N Sex differences in hemodynamic responses and long-term survival to optimal medical therapy in patients with pulmonary arterial hypertension. Heart Vessels. (2018) 33(8):939–47. 10.1007/s00380-018-1140-6 29441403 PMC6060798

[164] Kawut SM Archer-Chicko CL DeMichele A Fritz JS Klinger JR Ky B Anastrozole in pulmonary arterial hypertension. A randomized, double-blind, placebo-controlled trial. Am J Respir Crit Care Med. (2017) 195(3):360–8. 10.1164/rccm.201605-1024OC 27602993 PMC5328182

[165] Kawut SM Pinder D Al-Naamani N McCormick A Palevsky HI Fritz J Fulvestrant for the treatment of pulmonary arterial hypertension. Ann Am Thorac Soc. (2019) 16(11):1456–9. 10.1513/AnnalsATS.201904-328RL 31314997 PMC6945473

[166] Ghantous I Tlais M Khatib AE Ghantous G Maroun G Challita A The impact of Pde5 inhibitors on cardiovascular outcomes. Glob Cardiol Sci Pract. (2025) 2025(3):e202531. 10.21542/gcsp.2025.31 41346650 PMC12673857

[167] Cole A Kielbowski K Dach A Szulc J Bakinowska E Pawlik A. Endothelin as a treatment target in cardiovascular diseases: a recent step forward. Rev Cardiovasc Med. (2025) 26(12):45417. 10.31083/RCM45417 41524047 PMC12781005

[168] Anand V Garg S Duval S Thenappan T. A systematic review and meta-analysis of trials using statins in pulmonary arterial hypertension. Pulm Circ. (2016) 6(3):295–301. 10.1086/687304 27683606 PMC5019082

[169] Wilkins MR Ali O Bradlow W Wharton J Taegtmeyer A Rhodes CJ Simvastatin as a treatment for pulmonary hypertension trial. Am J Respir Crit Care Med. (2010) 181(10):1106–13. 10.1164/rccm.2009111-699Oc 20460548 PMC2874452

[170] Rysz-Gorzynska M Gluba-Brzozka A Sahebkar A Serban MC Mikhailidis DP Ursoniu S Efficacy of statin therapy in pulmonary arterial hypertension: a systematic review and meta-analysis. Sci Rep. (2016) 6:30060. 10.1038/srep30060 27444125 PMC4957081

